# Renal-tubular-mitochondrial sequentially targeted nanoagent breaks the vicious cycle of oxidative stress and mtDNA-driven inflammation in acute kidney injury therapy

**DOI:** 10.1186/s12951-026-04049-2

**Published:** 2026-01-24

**Authors:** Chenli Zhang, Ling Tan, Pengfei Yang, Lili Huang, Zeli Xiang, Lingshan Zhao, Ling Zhang, Jun Deng, Xiaohui Liao

**Affiliations:** 1 Department of Nephrology, The Second Affiliated Hospital, Chongqing Medical University, Chongqing, 400016 China; 2 Institute of Burn Research, Southwest Hospital, State Key Lab of Trauma and Chemical Poisoning, Army Medical University, Chongqing, 400038 China; 3 Department of nephrology, Second People’s Hospital of Yibin, Yibin, 644000 China

**Keywords:** Acute kidney injury, Cascade targeting, Renal tubular epithelial cells, Mitochondrial DNA, Nanocarriers

## Abstract

Acute kidney injury (AKI) is a life-threatening disorder that is responsible for 1.7 million deaths each year. Current treatment strategies, such as renal replacement therapy and supportive care, remain ineffective in reversing tubular damage in 20–50% of patients, largely due to their inability to promptly restore renal tubular epithelial cells function. The present study developed a cascade-targeted core-shell nanotherapeutic agent STMB with L-serine shell that enables precise kidney injury molecule 1-mediated delivery to damaged RTECs, while tannic acid core facilitates mitochondrial localization. Tannic acid continuously scavenges reactive oxygen species. By simultaneously clearing reactive oxygen species and preventing mitochondrial DNA leakage by STMB, this dual-channel intervention successfully terminates the self-amplifying cycle of oxidative damage and inflammatory signaling, resulting in therapeutic effects in both cisplatin-induced and ischemia-reperfusion AKI models. This strategy redefines AKI therapeutics by transitioning from passive symptom management to active cellular repair, which fills a critical void in clinical nephrology and opens new avenues for organ-protective nanomedicine development in critical care settings.

**Graphical Abstract:**

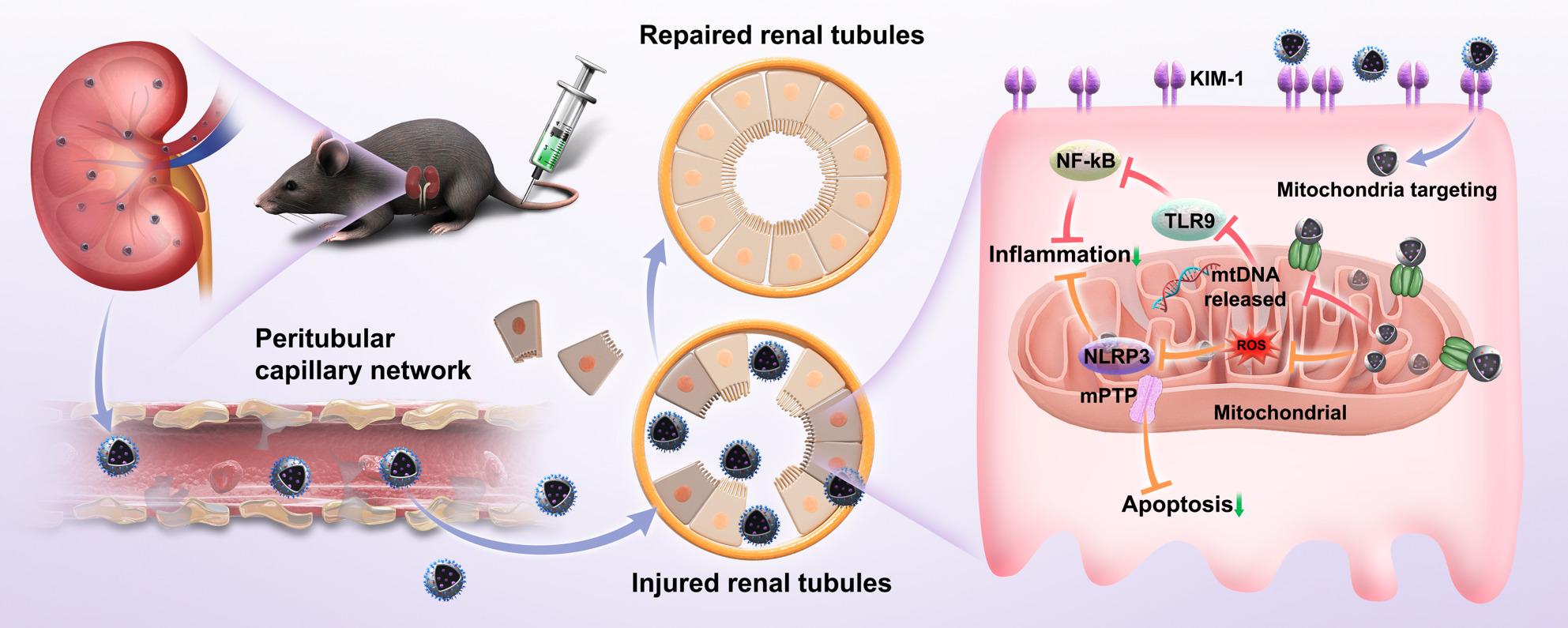

**Supplementary Information:**

The online version contains supplementary material available at 10.1186/s12951-026-04049-2.

## Introduction

Acute kidney injury (AKI) is a severe clinical condition characterized by rapid renal function deterioration. It affects approximately 13.3 million people worldwide annually and is associated with roughly 1.7 million deaths every year [1–3]. Current therapeutic approaches, including etiological intervention, supportive care, and renal replacement therapy, aim to manage complications, such as fluid overload, electrolyte imbalances, and acidosis, with the expectation of spontaneous repair of tubular epithelial cells [4, 5]. However, renal function remains irreversibly impaired in 20–50% of patients, and the mortality rate in severe cases exceeds 50% [6, 7]. This poor prognosis stems largely from the limitations of existing therapies in effectively halting ongoing cellular damage, particularly the persistent inflammatory injury within the renal tubules, which critically impedes the intrinsic repair processes. Therefore, there is a critical need to develop strategies that actively mitigate renal tubular inflammation and associated damage, thereby creating a conducive microenvironment for tissue repair in order to address the clinical challenges of AKI and improve long-term patient outcomes.

Recent efforts in AKI intervention have prioritized alleviating pathological damage to renal tubular epithelial cells (RTECs) by targeting key drivers of injury, such as oxidative stress and inflammation. Strategies employing antioxidants to scavenge reactive oxygen species (ROS) or delivering anti-inflammatory agents can provide transient relief from acute oxidative damage [8–11]. Although simply clearing ROS or employing anti-inflammatory agents can temporarily alleviate oxidative damage, it does not restore mitochondrial structural integrity. Moreover, the residual mitochondrial DNA (mtDNA) can still lead to the continuous pro-inflammatory factor secretion, which eventually results in inflammation amplification and cell death pathway reactivation. Alternative approaches attempt to directly stimulate RTECs proliferation via targeted delivery of pro-repair growth factors [12, 13]. Nevertheless, the effectiveness of this strategy is frequently compromised by a robust inflammatory influx of immune cells and the persistent ROS release within the tissue milieu [14, 15]. An effective strategy must fundamentally disrupt AKI’s self-sustaining injury cycle through coordinated intervention against both ROS accumulation and mtDNA-driven inflammation to halt progressive damage and enable intrinsic repair.

The abnormal mtDNA release is one of the core driving factors behind the impaired RTECs repair in AKI [15, 16]. Aberrantly released mtDNA activates the innate immune pathways upon RTECs damage, triggering persistent inflammatory signaling that directly exacerbates tubular injury and impedes cellular recovery. Critically, this mtDNA-driven inflammation suppresses the expression of key cyclins and reparative factors, inducing cell cycle arrest and perpetuating a state of cellular dysfunction [17]. Therefore, suppressing mtDNA release is essential to disrupt this pathological cascade and mitigate the sustained inflammatory blockade of intrinsic repair capacity. To achieve this goal, a delivery system with the following dual functions had to be developed: (1) Multi-level targeting ability, where the carrier must possess the capability of “renal enrichment-RTECs targeting-precise mitochondrial localization,” ensuring that the intervention molecules are efficiently enriched in the mitochondria of damaged RTECs. (2) Functional synergy, such that the carrier must integrate mtDNA release inhibition and ROS scavenging, controlling inflammation at its source to reduce further damage to RTECs and enabling reparative microenvironment restoration.

**Fig. 1:**
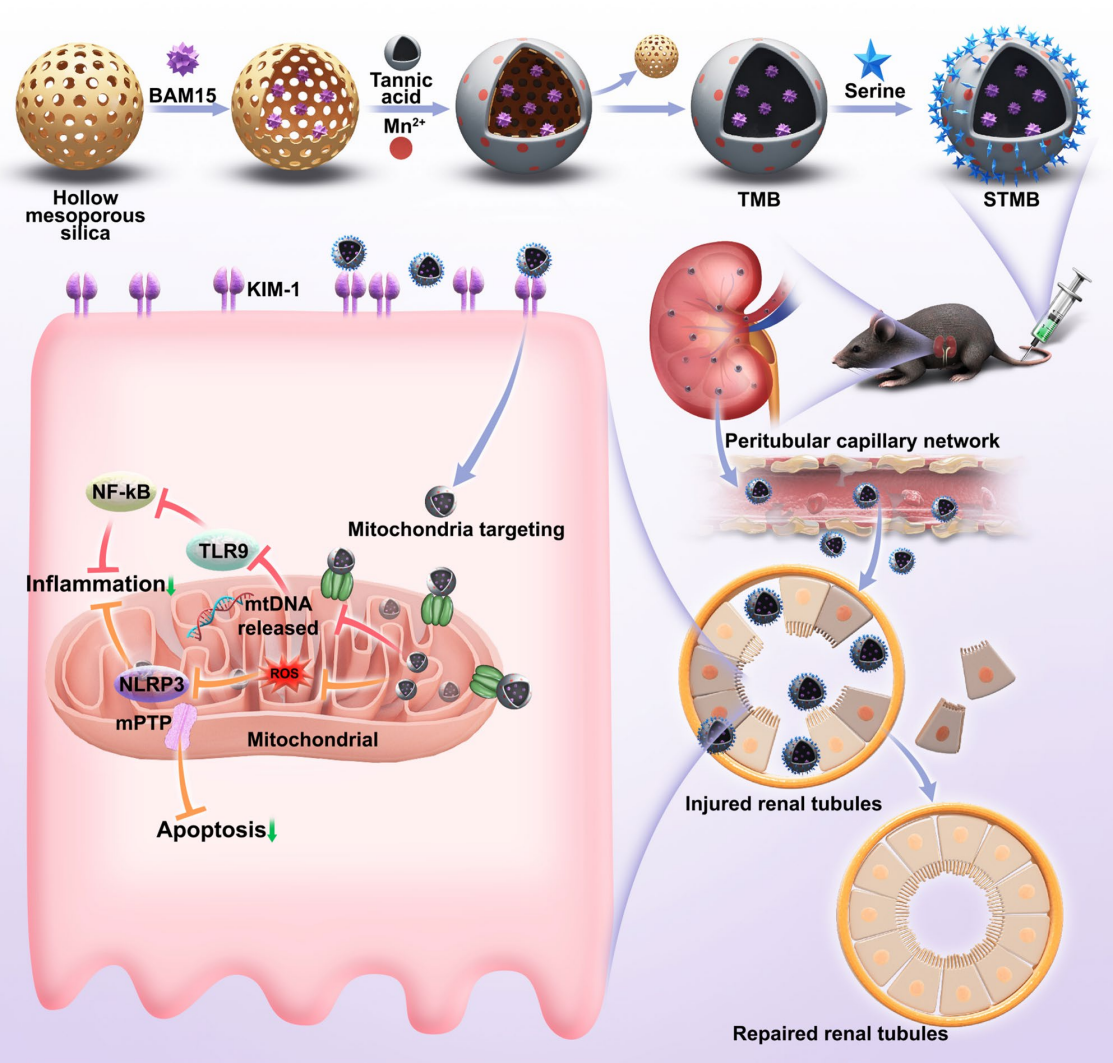
Schematic illustration showing the construction of cascade-targeted delivery system STMB and its therapeutic mechanism for alleviating acute kidney injury. STMB reaches the renal tubules due to the increase in peritubular capillary permeability in AKI. It achieves primary targeting by specifically binding RTEC KIM-1 receptor via STMB L-serine. It actively targets damaged mitochondria through strong interactions between STMB tannic acid and voltage-dependent anion-selective channels and outer membrane transport proteins on the mitochondrial membrane, achieving secondary targeting. STMB then simultaneously clears ROS, reduces mitochondrial DNA leakage, alleviates persistent inflammatory responses and apoptosis, improves RTEC damage, and promotes RTECs repair

The present study designed a new multi-functional cascade targeted nanocarrier system STMB, which precisely targets RTECs mitochondria, alleviates inflammatory response, protects mitochondrial function, and reduces apoptosis, creating more favorable conditions for kidney repair and promoting kidney tissue repair and regeneration. The core of the system is formed by the tannic acid (TA)-manganese (Mn) chelate complex co-loaded with the mitochondrial uncoupler N5,N6-bis(2-fluorophenyl)(1,2,5)oxadiazolo(3,4-b)pyrazine-5,6-diamine (BAM15), while the shell is modified with L-serine (Ser) (Fig. 1). This design fully exploits the synergistic advantages of the core-shell structure, where STMB enters the renal tubule from the peritubular capillaries through the basolateral membrane [18]. The Ser-modified shell specifically binds to the kidney injury molecule 1 (KIM-1) receptor, which is highly expressed on RTECs in AKI, achieving “kidney to RTECs” primary targeting [19]. The TA in the core actively targets damaged mitochondria due to its affinity for mitochondria through strong interactions with voltage-dependent anion-selective channel (VDAC) and translocase of outer membrane (TOM) proteins on mitochondrial membranes, thereby realizing “cell to mitochondria” secondary targeting [20]. TA continuously scavenges ROS [21], releasing BAM15 to stabilize the mitochondrial membrane potential (MMP), prevents mtDNA leakage, and synergistically clears ROS production [22]. In two major experimental models that effectively simulate the pathophysiological mechanism of AKI (cisplatin (Cis)-induced and Ischemia-reperfusion (I/R)), STMB significantly reduces serum creatinine (SCr) levels, scavenges ROS, reduces mtDNA leakage, ameliorates RTECs injury, and promotes RTECs repair. This work has synergistically achieved mtDNA release blockade and ROS scavenging through the “three-level targeted logic”, breaking away from the traditional model of “poor effective single-target intervention” in AKI treatment, realizing damage suppression and repair activation and providing a new paradigm for nanomedicine design for organ injury repair.

## Results

### STMB characterization

**Fig. 2:**
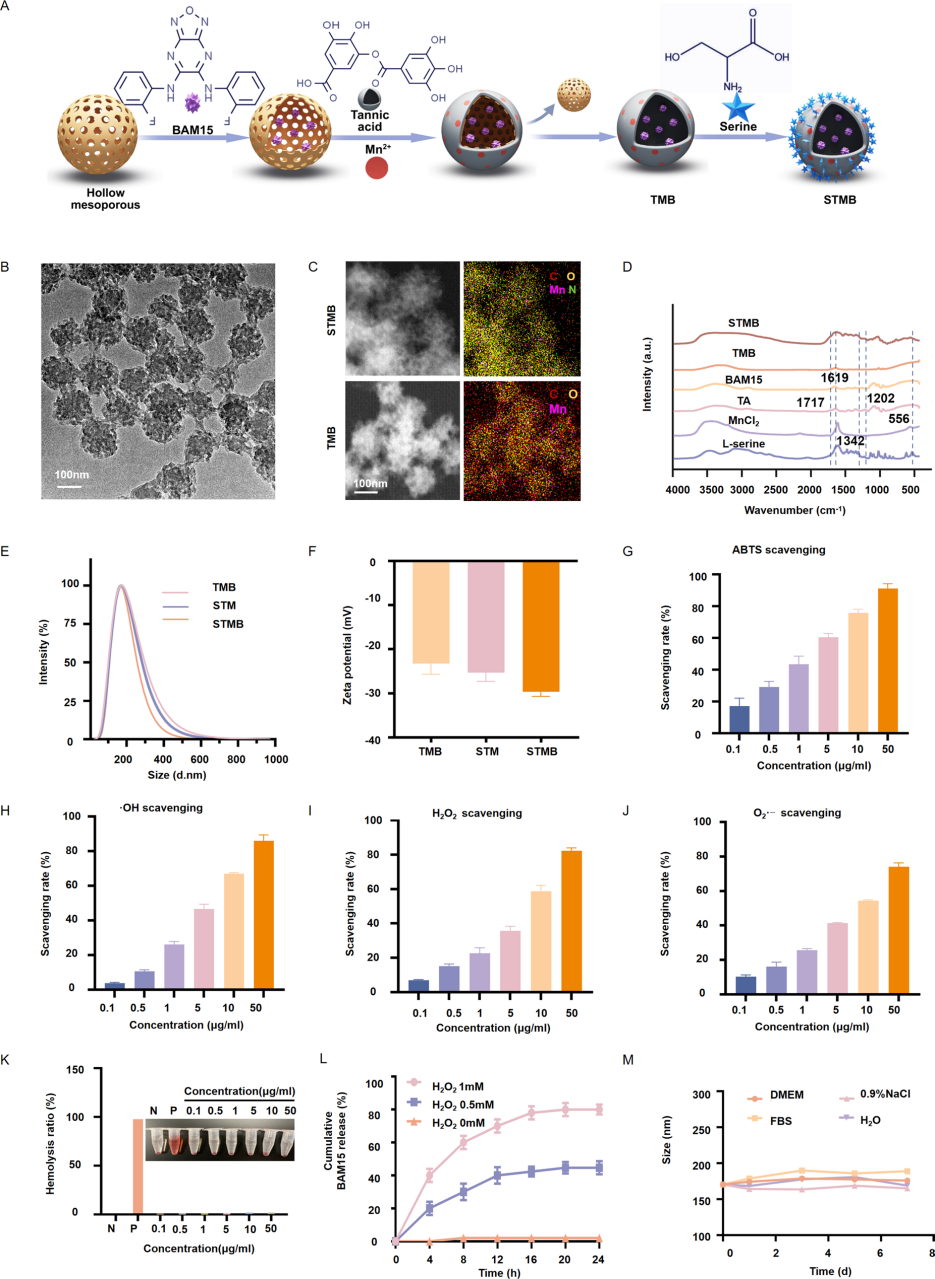
STMB characterization. (**A**) Synthetic route for STMB generation. (**B**) Representative TEM images of STMB. (**C**) Elemental mapping images of TMB and STMB, respectively(upper is STMB, down is TMB). (**D**) FTIR spectra for STMB, TA, BAM15, MnCl₂ and L-serine. (**E**) Hydrodynamic diameter distribution of STM, TMB, and STMB. (**F**) Zeta potential of STM, TMB, and STMB. Free radical scavenging capacity of STMB on (**G**) ABTS, (**H**) •OH, (**I**) H₂O₂, and (**J**) O₂•−. (**K**) Hemolysis assay indicated that STMB had good biosafety. Water served as positive control and saline solution served as negative control. (**L**) Cumulative release of BAM15 from STMB at different times in H₂O₂ (0 mM, 0.5 mM, and 1 mM). (**M**) Hydrodynamic diameter changes of STMB in different media for 7 days. *n* = 5, *p < 0.05; **p < 0.01; and ***p < 0.001

BAM15 was loaded using mesoporous silicon dioxide and mixed with TA and Mn chloride tetrahydrate to initiate the self-assembly process to form water-soluble nanoparticles (NPs) (Fig. 2A). NPs with varying amounts of BAM15 were prepared while maintaining fixed ratios of TA and Mn. These NPs were used to identify the formulation with a BAM15:TM (TA + Mn) mass ratio of 16:1 as one exhibiting maximal drug loading capacity (4.84 ± 0.04) and efficiency (75.37 ± 0.18; Table S1), which was consequently selected for subsequent studies. STMB was fabricated by conjugating Ser to the NP surface via an carbodiimide chemistry(EDC/NHS) mediated amidation reaction, where the α-carboxyl group (-COOH) of Ser forms a covalent bond with the activated amino groups (-NH_2_) on TMB [23]. The STMB NPs demonstrated uniform monodispersity with a dry diameter of 170 nm determined using transmission electron microscopy (TEM; Fig. 2B). Elemental mapping revealed the presence and distribution of carbon (C), oxygen (O), nitrogen (N), and manganese (Mn) elements in STMB. The mapping analysis showed their spatial distribution positions, and the merge plot showed that the above elements could overlap perfectly, indicating the successful STMB synthesis. In contrast, TMB lacked nitrogen (N) in its elemental composition (Fig. 2C). Fourier infrared detection revealed that STMB existed at 1,717 cm^− 1^, corresponding to C = O stretching vibration in -COOH, 1,619 cm^− 1^, corresponding to C = C and C = N stretching vibration, 1,342 cm^− 1^, corresponding to C-H bending vibration, 1,202 cm^− 1^, corresponding to C-O stretching vibration, and 556 cm^− 1^, corresponding to O-H bending vibration (Fig. 2D). Dynamic and electrophoretic light scattering measurements revealed that STMB had a diameter of 170.49 nm with a zeta potential of −29.61 mV, while STM measured 173.19 nm with a zeta potential of −25.29 mV. TMB was 173.39 nm in size and − 23.22 mV (Fig. 2E, F). Although the three types exhibited comparable particle sizes, the elevated absolute zeta potential of STMB indicated superior colloidal stability.

Various types of AKI pathophysiological processes are closely linked to excessive reactive oxygen and nitrogen species (RONS). Thus, the antioxidative properties of nanomaterials play a crucial role in shielding kidneys from oxidative harm [24, 25]. The RONS scavenging capacity of STMB was systematically examined. Specifically, its ability to scavenge RONS using the 2,2′-azinobis-(3-ethylbenzthiazoline6-sulphonate(ABTS) probe was assessed, which was widely recognized for nitrogen-based free radical scavenging evaluation. STMB was applied at concentrations ranging from 0.1 to 50 µg mL^− 1^, corresponding to an escalating elimination rate from 17% to 85%, respectively (Fig. 2G). Additionally, STMB’s capacity to scavenge representative ROS by hydroxyl radical (•OH), hydrogen peroxide (H_2_O_2_), and superoxide anion radical (O_2_^• −^) was investigated (Fig. 2H–J). STMB showed significant clearance ability for a variety of RONS. The clearance efficiency increased with the increase in concentration, indicating that it may have a strong and effective protective potential in AKI when RONS burst. The hemolytic activity of STMB remained below 5% across all replicates at the highest tested concentration of 50 µg mL^− 1^ (Fig. 2K), indicating that STMB exhibits favorable blood compatibility. The disintegration characteristics of STMB in H_2_O_2_ led to the responsive release of BAM15, which was due to the obvious morphological collapse of STMB treated with H_2_O_2_ (Fig. S1). The release rate of BAM15 under H_2_O_2_ was 82.0%, 44%, and 2.2% at 1 mM, 0.5 mM, and 0 mM after 24 h, respectively (Fig. 2L).

The structural integrity and colloidal stability of nanomaterials are critical determinants of their preservation and successful in vivo applications. To systematically evaluate these characteristics, STMB was dispersed in four physiologically relevant media (deionized water, 0.9% NaCl, Dulbecco’s Modified Eagle Medium (DMEM), and fetal bovine serum (FBS)) over the course of seven days. Dynamic light scattering analysis revealed neither significant particle aggregation nor notable changes in hydrodynamic diameter, confirming its exceptional stability under all tested conditions (Fig. 2M).

### *In vitro* ROS scavenging, mitochondrial protection, and apoptosis inhibition capacity of STMB

**Fig. 3:**
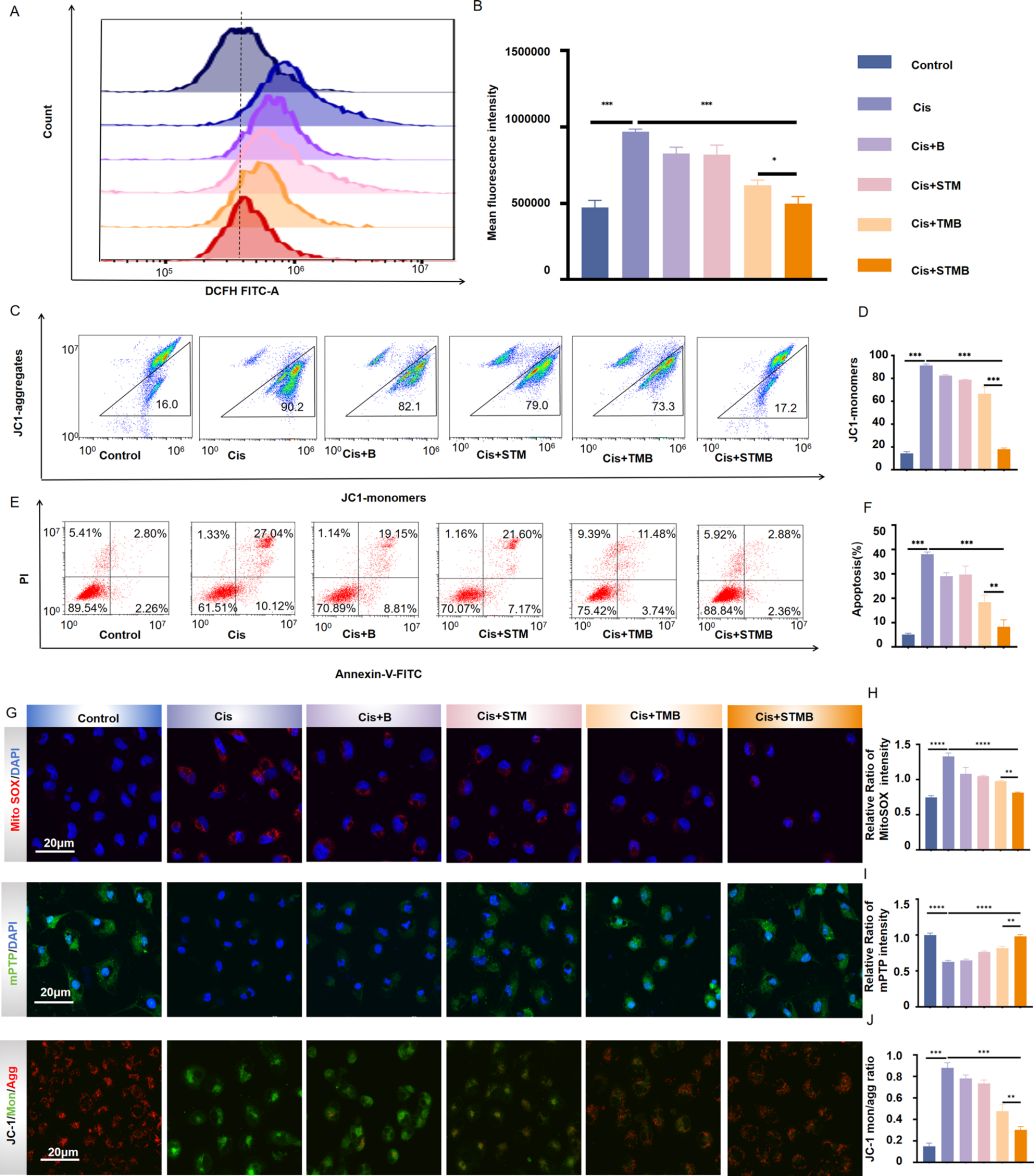
In vitro ROS scavenging, mitochondrial protection, and apoptosis inhibition of STMB. (**A**) Intracellular ROS levels determined by flow cytometry and (**B**) their quantitative analysis. (**C**) MMP of HK-2 cells assessed using flow cytometry using JC-1 after different treatments and (**D**) their quantitative analysis for JC1-monomers. (**E**) Apoptosis assays based on flow cytometry analysis and (**F**) their quantitative analysis for HK-2 apoptosis ratio after different treatments. (**G**) Confocal image analysis for Mito Sox, mPTPs, and JC-1. (**H**–**J**) Quantitative analysis for Mito Sox, mPTPs, and JC-1. *n* = 3, *p < 0.05; **p < 0.01; and ***p < 0.001

Using the HK-2 cells as a physiologically relevant model of drug-induced nephrotoxicity, STMB (1 µg mL^− 1^) demonstrated potent cytoprotection against Cis via dual mechanisms: mitochondrial functional restoration and apoptotic cascade suppression. Flow cytometric assessment revealed a significant increase in ROS within the cisplatin-treated group compared to the control group. In contrast, all treatment groups demonstrated a pronounced decrease in ROS level. Notably, the STMB group displayed the most significant decrease (*p* < 0.001 vs. Cis), strongly indicating its superior ROS scavenging ability (Fig. 3A, B). 5,5′,6,6′-Tetrachloro-1,1′,3,3′-Tetraethyl-Imidacarbocyanine iodide (JC-1) monomer levels markedly increased when the MMP decreased. Consistent with this, Cis-treated HK-2 cells showed a dramatic rise in monomer levels (*p* < 0.001 vs. control). By comparison, all treatment groups exhibited a reduction in monomer levels, with the STMB group demonstrating the most pronounced efficacy, nearly returning to baseline concentrations (*p* < 0.001 vs. Cis). The JC-1 monomer/aggregate ratio was also significantly lowered (*p* < 0.001 vs. Cis), further confirming the MMP recovery (Fig. 3C, D). In addition, flow cytometry demonstrated the protective effects of each treatment against Cis-induced apoptosis. As expected, the Cis group exhibited a significantly highest apoptosis rate (*p* < 0.001 vs. control), whereas all treatment groups attenuated apoptosis. These results strongly suggest that the treatments conferred protection by effectively reducing intracellular ROS levels (Fig. 3E, F). After entering the mitochondria, MitoSOX Red assay further validated these findings. Confocal microscopy revealed that the Cis group displayed the most intense red fluorescence, while all treatment groups showed obviously weaker signals. The STMB group exhibited a more pronounced reduction in fluorescence level than the TMB group (*p* < 0.01), providing compelling evidence that it efficiently and selectively scavenges mitochondrial ROS (Fig. 3G top, H). Excessive mROS triggers the opening of mitochondrial permeability transition pores (mPTPs) [25], which can be detected using a calcium chlorophyll acetate methyl ester probe. Confocal microscopy analysis revealed a striking contrast in mPTPs activity between groups. While the Cis group exhibited a nearly complete absence of green fluorescence, all of the treatment groups demonstrated a dose-dependent recovery of blue-green fluorescence. Most notably, the STMB group showed the most pronounced residual green fluorescence signal, providing direct visual and quantitative evidence of mPTPs closure. This finding stands in sharp contrast to that observed in the Cis group (*p* < 0.001), where mPTP-driven fluorescence quenching was characteristically severe, further confirming STMB’s ability to preserve mitochondrial integrity (Fig. 3G middle, I). Changes in MMP are indicative of the early apoptosis stages [26]. A comparison of JC-1 polymer (red) enrichment in mitochondria of treated cells to the diffuse monomeric (green) signals in the AKI groups confirmed MMP preservation (Fig. 3G bottom). Fluorescence confocal microscopy can also reflect MMP through fluorescence staining using JC-1 probes. JC-1 fluorescence shifts from red to green when MMP decreases. Confocal microscopy assessment of HK-2 cells exposed to Cis showed mitochondrial impairment, as indicated by a marked decline in red fluorescence (JC-1 aggregates) alongside a rise in green fluorescence (JC-1 monomers). Strikingly, the STMB treatment exhibited the most robust protective effect among all interventions, demonstrating the highest degree of red fluorescence preservation and the most pronounced green fluorescence intensity suppression. These findings provide compelling evidence that STMB more effectively restores MMP (*p* < 0.01 vs. TMB) and attenuates Cis-induced apoptosis, highlighting its superior cytoprotective capacity (Fig. 3G bottom, J).

### Hierarchical STMB targeting in the kidney, renal tubular epithelial cells, and mitochondria

**Fig. 4:**
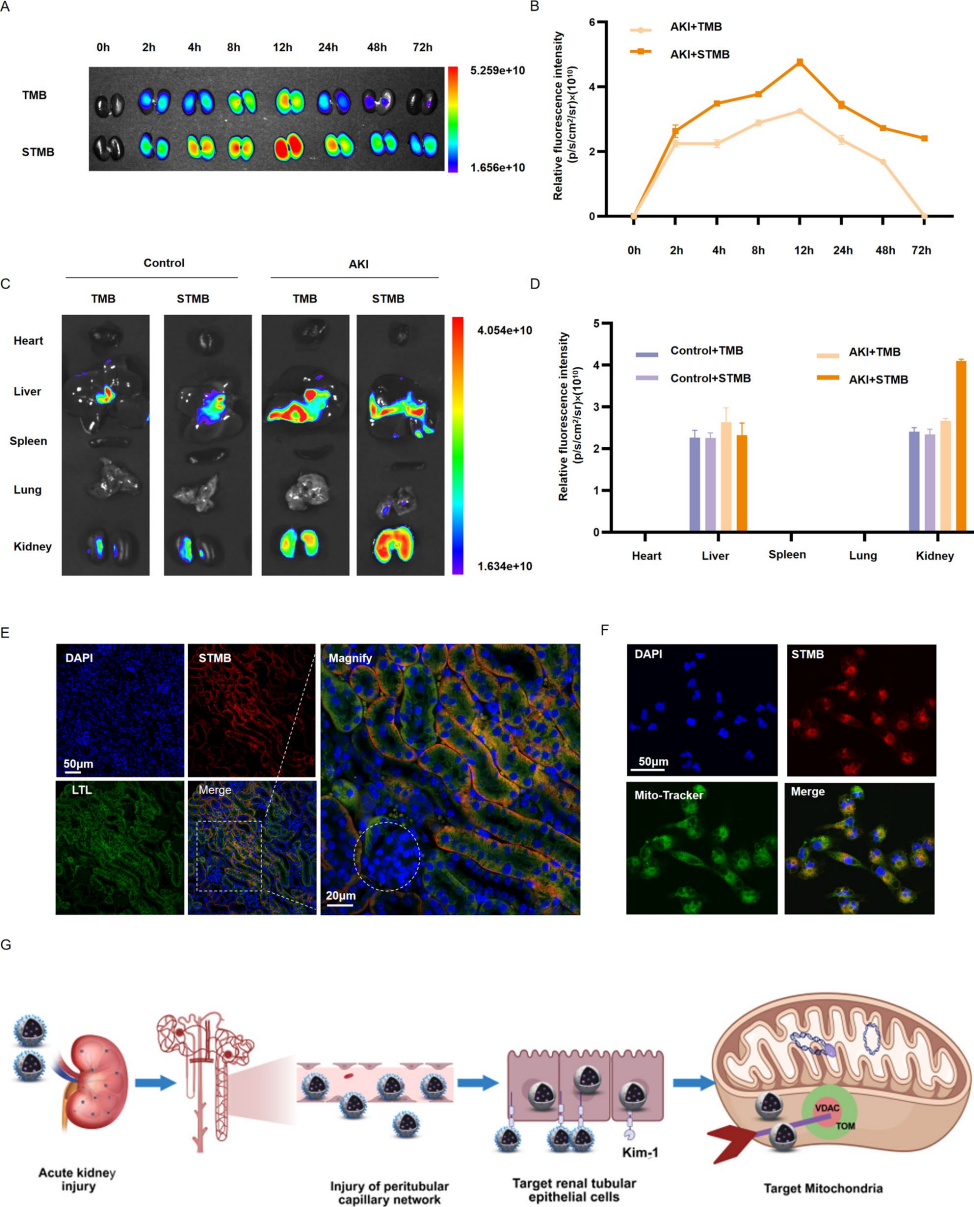
Hierarchical targeting of STMB in the kidney, renal tubular epithelial cells, and mitochondria. (**A**) Representative fluorescence images of mouse kidney tissues 0, 2, 4, 8, 12, 24, 48, and 72 h following administration of TMB or STMB after intraperitoneal injection of cisplatin and (**B**) quantitative analysis of their relative intensity. (**C**) Representative fluorescence images of major organs in mice treated with STMB and TMB at 12 h and (**D**) their relative quantitative intensity analysis. (**E**) STMB (red) and LTL (green) co-localization in cisplatin-induced AKI kidney tissues. White dotted line represents glomerulus. (**F**) Confocal microscopy images showing STMB co-localization with mitochondria. Mitochondria tracker labels mitochondria (green), DiI labels STMB (red). (**G**) Schematic illustration showing cascade-targeting mechanism of the nanotherapeutic system in AKI. *n* = 3, *p < 0.05; **p < 0.01; and ***p < 0.001

Water soluble Cyanine 5 (Cy5) fluorescent dye was utilized for labeling in order to investigate the cascade targeting of STMB. First, 100 µL of Cy5-labeled TMB or STMB was administered to mice with Cis-induced AKI via the tail vein. Fluorescence images were acquired at multiple time points (2, 4, 8, 12, 24, 48, and 72 h post-treatment). AKI model mice with saline injection were utilized as the control. NP biodistribution in the kidney was also examined. The findings revealed that NPs were initially concentrated in the kidney within 2 h, with fluorescence intensity progressively increasing over time and peaking at 12 h before gradually declining. Notably, the STMB group demonstrated prolonged renal retention compared to the TMB group, with obvious accumulation still observed at the 72-h mark (Fig. 4A, B). Notably, NP accumulation was significantly enhanced in the kidneys of AKI model mice relative to that in the control animals, with STMB NPs exhibiting a pronounced targeting preference for Cis-injured renal tissue (Fig. 4C). The NP intensity in the livers of control mice was less notable than that in the AKI group, which may be due to congestion and increased permeability of hepatic vessels during AKI leading to NP accumulation in the liver. STMB accumulation in the kidney was confirmed to be 1.54 times that of the TMB group, which may play a role in relieving AKI (Fig. 4D). The concentration of manganese ions in the cerebrospinal fluid (CSF) of mice 12 h after the injection of STMB was detected by mass spectrometry. The results showed that intravenous injection of STMB did not increase the concentration of manganese ions in the CSF, indicating that STMB cannot cross the blood-brain barrier (Fig. S2).Taken together, these findings indicate that STMB achieves strong renal retention, a property that likely underlies its therapeutic potential.

Since RTECs are the most numerous and represent the highest percentage of cells in the kidney tissue, their injury and death are the most critical in the decline of renal function. This dysfunction is the initiating event in AKI and the key to AKI repair [27]. To further evaluate the specific biodistribution within kidney tissue and the renal tubular targeting capability of STMB, renal sections were harvested from AKI mice 12 h post-administration of Cy5-STMB. Microscopic fluorescence images were subsequently obtained. The Cy5-labeled STMB fluorescence was primarily observed within the renal tubular regions, as evidenced by Lotus tetragonolobus lectin labeling, the Pearson correlation coefficient was 0.70 (Fig. 4E, S3). This finding underscores the targeting ability of STMB toward renal tubules. Therefore, the accumulation of STMB within these tubules suggests its potential as an effective drug carrier for AKI therapy.

Organelle localization was evaluated using Cy5-labeled NPs. STMB with Cy5 was incubated with HK-2 cells. Confocal microscopy analysis indicated that Cy5-labeled STMB was internalized by HK-2 cells after 2 h of incubation with the Cis treatment. An obvious overlap in fluorescence was observed between the STMB group and MitoTracker (green), the Pearson correlation coefficient was 0.85, indicating that STMB has the capability to selectively target mitochondria (Fig. 4F, S4).

STMB leverages peritubular capillary permeability in AKI to reach the renal tubules and binds to the KIM-1 receptor on renal RTECs via its Ser component, achieving RTECs-specific binding. Utilizing the TA contained within STMB, this agent subsequently achieves active mitochondrial targeting by influencing its strong interactions with the VDAC proteins on the mitochondrial membrane, thereby enabling precise mitochondrial STMB localization (Fig. 4G, S5, and S6).

### Therapeutic STMB efficacy in Cis-mediated AKI mouse model

**Fig. 5:**
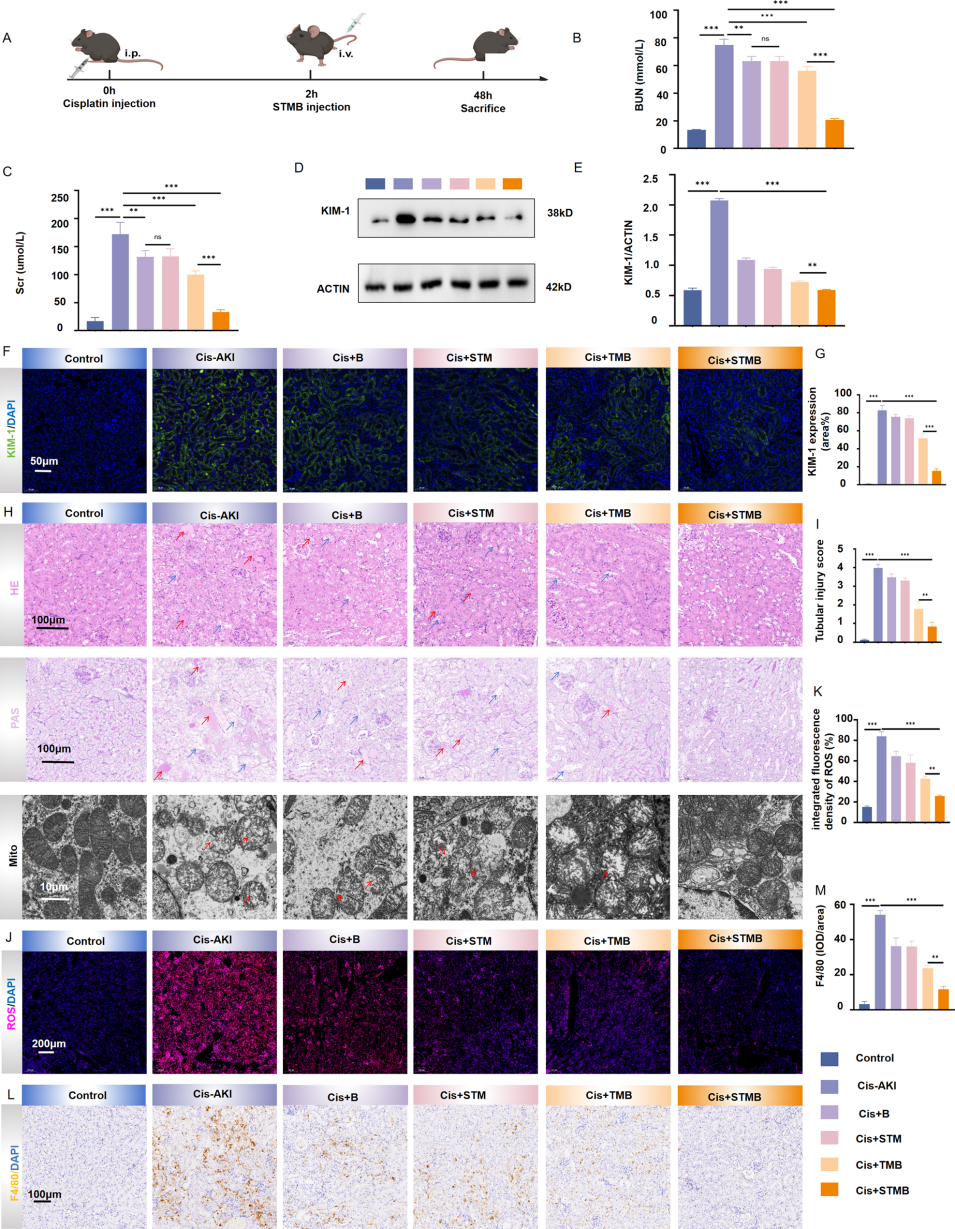
STMB scavenges excess ROS, protects mitochondria, reduces renal injury, and decreases macrophage infiltration in mouse models of cisplatin (Cis)-mediated AKI. (**A**) Schematic illustration depicting the establishment of Cis-mediated AKI mouse models and subsequent therapeutic regimen. (**B**) Renal function evaluation in terms of (**B**) BUN and (**C**) SCr levels in Cis-AKI mice after various treatments for 48 h. (**D**) Western blot results for KIM-1-associated protein expression in kidney tissue, where β-actin was used to normalize protein content, and (**E**) quantitative analysis. (**F**) Representative images of KIM-1 immunofluorescence staining in the kidney and (**G**) its quantitative analysis. (**H**) Representative images of Cis-AKI renal tissue stained with H&E and PAS. Red arrow represents fiber casting, and blue arrow represents brush-like edge of the proximal tubule and the exposed tubule basement membrane. TEM analysis of mitochondrial morphology. Red arrows show mitochondrial cristae fragmentation. (**I**) Quantitative analysis of tubular injury score in different treatment groups stained with H&E. (**J**) ROS levels in kidney tissues from different treatment groups stained with DHE and (**K**) quantitative analysis of ROS in immunofluorescence staining in the kidney. (**L**) Representative IHC staining results and (**M**) quantitative analysis of F4/80 in the kidneys. *n* = 3, *p < 0.05; **p < 0.01; and ***p < 0.001

Cis is a widely used chemotherapeutic agent. However, its application can trigger AKI. To investigate the potential protection effect of STMB on Cis-associated AKI, a Cis-induced AKI mouse model was established and STMB (10 mg kg^− 1^) was administered intravenously for potential intervention (Fig. 5A). Mice with AKI exhibited markedly increased levels of SCr and blood urea nitrogen (BUN), while control animals retained values within the normal range (*p* < 0.001), confirming the successful establishment of Cis-induced AKI mouse model. However, these levels in the STMB group remained close to those in the control group, indicating an improvement in renal function (Fig. 5B, C). The transmembrane protein KIM-1 is primarily expressed in the proximal tubule. It serves as a receptor for phosphatidylserine on apoptotic cells and is a well-established biomarker of renal damage, contributing significantly to Cis-induced cell damage [28–30]. Western blotting (WB) and the corresponding densitometry quantification in the Cis-induced AKI model verified a marked upregulation of KIM-1 expression in the AKI group following various treatment regimens (*p* < 0.001 vs. control), while showing decreases across all treatment groups, with the most significant reduction observed in the STMB group (*p* < 0.001 vs. Cis-AKI; Fig. 5D, E). In vivo evaluation using fluorescent dyes revealed enhanced green fluorescence expression in AKI renal tissues compared to that in the control group. Green fluorescence expression decreased across all treatment groups, with the STMB group exhibiting the weakest response. The STMB treatment group demonstrated a notable decrease in KIM-1 expression relative to that in the Cis-induced AKI model group (*p* < 0.001 vs. Cis-AKI; Fig. 5F, G). Histological examination showed compromised renal basement membranes and a lack of brush borders in AKI mice compared to the control mice (Fig. 5H top, middle). Quantitative assessment of renal tubular injury based on H&E-stained sections indicated that the STMB treatment group exhibited markedly reduced scores compared to those in the TMB group (*p* < 0.01; Fig. 5I). The mitochondrial morphology in tubular epithelial cells in Cis-induced AKI was examined utilizing TEM, which confirmed outer mitochondrial membrane rupture and a decrease or absence of mitochondrial cristae in these cells. The mitochondrial outer membrane rupture of STMB cells was reduced in all treatment groups, while the mitochondrial crest recovery was the most obvious (Fig. 5H bottom). Immunofluorescence analysis results revealed a significant elevation in ROS levels within the AKI group (*p* < 0.001 vs. control), while the ROS infiltration was improved in the treatment group, especially the STMB intervention group (*p* < 0.01 vs. TMB; Fig. 5J, K). Excessive macrophage infiltration and activation can promote the damage, death, and abnormal repair of RTECs when kidney injury occurs, thus exacerbating renal damage during AKI [31]. Immunohistochemical results from this study demonstrated decreased F4/80 expression in renal tissues after STMB treatment relative to all other experimental groups following the induction of AKI (*p* < 0.001 vs. Cis-AKI; Fig. 5L, M). It was further demonstrated that STMB can reduce RTECs injury and alleviate AKI by inhibiting macrophage infiltration.

### Therapeutic STMB mechanisms in AKI

**Fig. 6:**
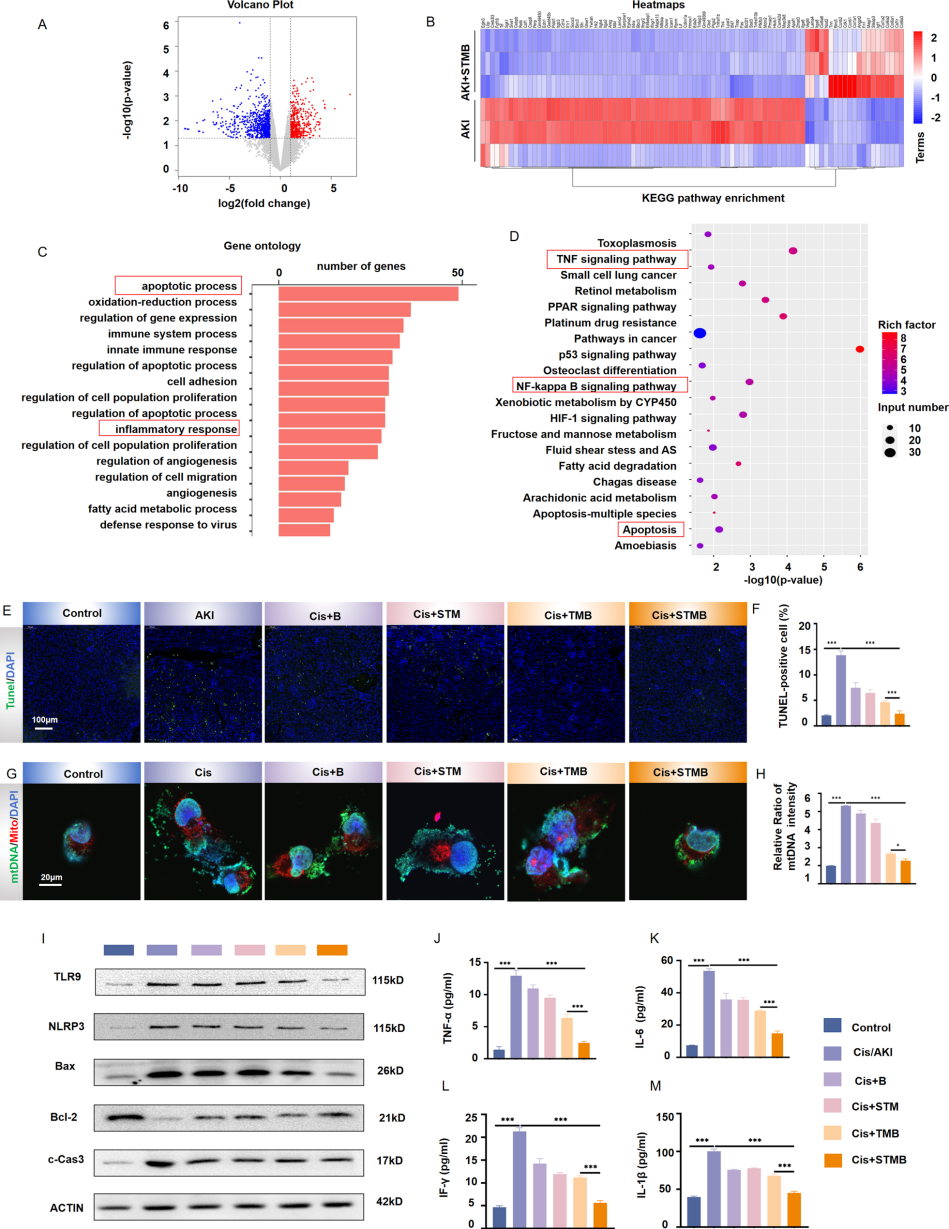
Therapeutic STMB mechanisms in AKI. (**A**) Volcano plots showing up- (red) and down-regulated (blue) genes in STMB-treated AKI mice. (**B**) Heatmaps of typical differentially expressed genes between the AKI + STMB and AKI + PBS groups. (**C**) Gene Ontology analysis of molecular STMB functions. (**D**) KEGG pathway enrichment analysis of the identified differentially expressed genes. Top 20 most significantly enriched pathways are shown. (**E**) Apoptotic cells were evaluated using TUNEL staining in kidney tissues after STMB injection. Representative TUNEL staining images showing blue nuclear staining by DAPI and green TUNEL staining and (**F**) their qualitive analysis in terms of TUNEL^+^ cells. Scale bar: 100 μm. *n* = 3. (**G**) Mitochondrial DNA release detected by fluorescence confocal microscopy. (**H**) Relative ratio of mtDNA intensity analysis. *n* = 3. (**I**) WB analysis of TLR9, NLRP3 and apoptosis-associated protein expression in kidney tissue. *n* = 3. (**J**–**M**) Detection of inflammatory marker expression levels in mouse serum using ELISA. *n* = 5, *p < 0.05; **p < 0.01; and ***p < 0.001

Transcriptomic analysis was conducted to further elucidate the potential therapeutic mechanisms of STMB in AKI. Kidney tissues from both the AKI and STMB treatment groups were examined, revealing significant differences in the expression of 1,242 genes, comprising 519 up-regulated and 723 down-regulated genes (Fig. 6A). The heat map of differentially expressed proteins indicated a considerable impact of STMB treatment on kidney gene expression (Fig. 6B), suggesting that STMB may effectively mitigate AKI. A subsequent functional enrichment analysis using the Gene Ontology database highlighted a strong correlation between apoptosis and inflammatory response in relation to the therapeutic mechanism of STMB (Fig. 6C). Pathway enrichment analysis using the Kyoto Encyclopedia of Genes and Genomes database indicated a strong correlation between STMB’s therapeutic effects and key biological pathways (Fig. 6D), highlighting its involvement in both the TNF/NF-κB signaling pathways and apoptosis.

### Suppressing STMB apoptosis and inflammation in AKI *via* mtDNA-TLR9/NLRP3 axis

The RTECs mitochondria become dysfunctional in the early AKI stages, leading to ATP deficiency, excessive ROS production, and further renal function deterioration. ROS overproduction initiates apoptotic pathways via multiple mechanisms, such as by inducing mitochondrial damage and promoting inflammatory cytokine secretion. This process initiates a feedback loop, where oxidative stress and chronic inflammation mutually reinforce each other, leading to escalated ROS production and exacerbating apoptotic and necrotic cellular damage [1, 32]. Terminal Deoxynucleotidyl Transferase Mediated dUTP Nick-End Labeling (TUNEL) staining results demonstrated that STMB attenuated apoptosis following AKI (*p* < 0.001 vs. Cis-AKI; Fig. 6E, F). Confocal imaging revealed that STMB treatment substantially suppressed mtDNA release after AKI induction, in contrast to the outcomes detected in the remaining intervention groups(*p* < 0.05 vs. TMB; Fig. 6G, H). WB analysis further indicated that each treatment led to a downregulation of Bax and an upregulation of BCL-2 expression following AKI induction (Figs. 6I, S7, and S8), with a subsequent reduction in cleaved caspase-3 expression level (Figs. 6I, S9). Of all treatment groups, the STMB group demonstrated the most significant restoration of these apoptotic markers compared to the AKI group (*p* < 0.001). Notably, ROS facilitated mtDNA leakage through various mechanisms [33]. Given that mtDNA contains numerous CpG islands, its release from necrotic cells can be recognized by the CpG DNA receptor TLR9. This recognition event subsequently triggers the TNF/NF-κB signaling cascade, leading to the transcriptional activation of numerous inflammatory cytokine genes, notably tumor necrosis factor-α (TNF-α) and interleukin-6 (IL-6) [34]. Furthermore, NLRP3 has been implicated in sensing ischemic damage that exacerbates inflammation, with its inhibition demonstrating protective effects in a mouse model of AKI [35]. Moreover, WB results revealed that the STMB treatment led to a significant downregulation of TLR9 and NLRP3 expression in the mouse model (*p* < 0.001 vs. Cis-AKI; Figs. 6I, S10, and S11). Enzyme-linked immunosorbent assay (ELISA) was employed to further elucidate the inflammatory response and quantify cytokines in mouse serum. This analysis showed that the STMB group exhibited a marked reduction in inflammatory markers relative to the control group, establishing its superior efficacy in lowering the levels of IL-6, TNF-α, interferon-gamma (IFN-γ), and interleukin-1 beta (IL-1β) (*p* < 0.001 vs. Cis-AKI; Fig. 6J–M). We simultaneously conducted the ELISA for anti-inflammatory factors, the results showed that the levels of IL-10 and TGF-β in the STMB group were significantly lower than those in the CIS-AKI group, indicating the beneficial anti-inflammatory effect of STMB **(**Figs. S12-13).

STMB alleviated the infiltration of macrophages in renal tissue. Macrophage polarization dictates the balance between inflammatory exacerbation and functional tissue repair after injury [36]. The aforementioned inflammatory factors are predominantly produced by M1-type macrophages, which can aggravate renal injury. Conversely, the repolarization of pro-inflammatory M1 macrophages toward an anti-inflammatory M2 state in the renal microenvironment contributes to tissue regeneration and functional recovery [37, 38]. Building on our prior findings, it was hypothesized that STMB could facilitate the M1-to-M2 phenotypic transition in macrophages. To test this hypothesis, macrophage polarization status was assessed through immunofluorescence staining targeting the M1 marker CD86 and the M2 marker CD206. Pronounced red fluorescence corresponding to CD86 was observed, while green fluorescence representing CD206 was diminished in the AKI group, which was indicative of M1 macrophage polarization (Fig. S14). In contrast, STMB treatment resulted in attenuated red fluorescence for CD86 alongside enhanced green fluorescence for CD206 staining, thereby confirming the promotion of M1-to-M2 polarization by STMB.

In summary, the therapeutic mechanism of STMB in AKI involves a multi-faceted approach targeting mitochondrial dysfunction and its downstream inflammatory cascades. By preserving mitochondrial integrity, STMB reduces ROS overproduction and prevents mtDNA leakage and subsequent TLR9/NF-κB pathway activation. Additionally, STMB exerts its anti-apoptotic effects by shifting the Bax/BCL-2 expression ratio toward a pro-survival state, while suppressing caspase-3 cleavage. The compound further mitigates inflammation by inhibiting NLRP3 inflammasome activation and reducing the production of pivotal cytokines TNF-α and IL-6. Collectively, these actions disrupt the cycle of oxidative stress, apoptosis, and inflammation, ultimately promoting renal repair and functional recovery in AKI.

### Therapeutic efficacy of STMB in I/R-mediated AKI mouse model

**Fig. 7:**
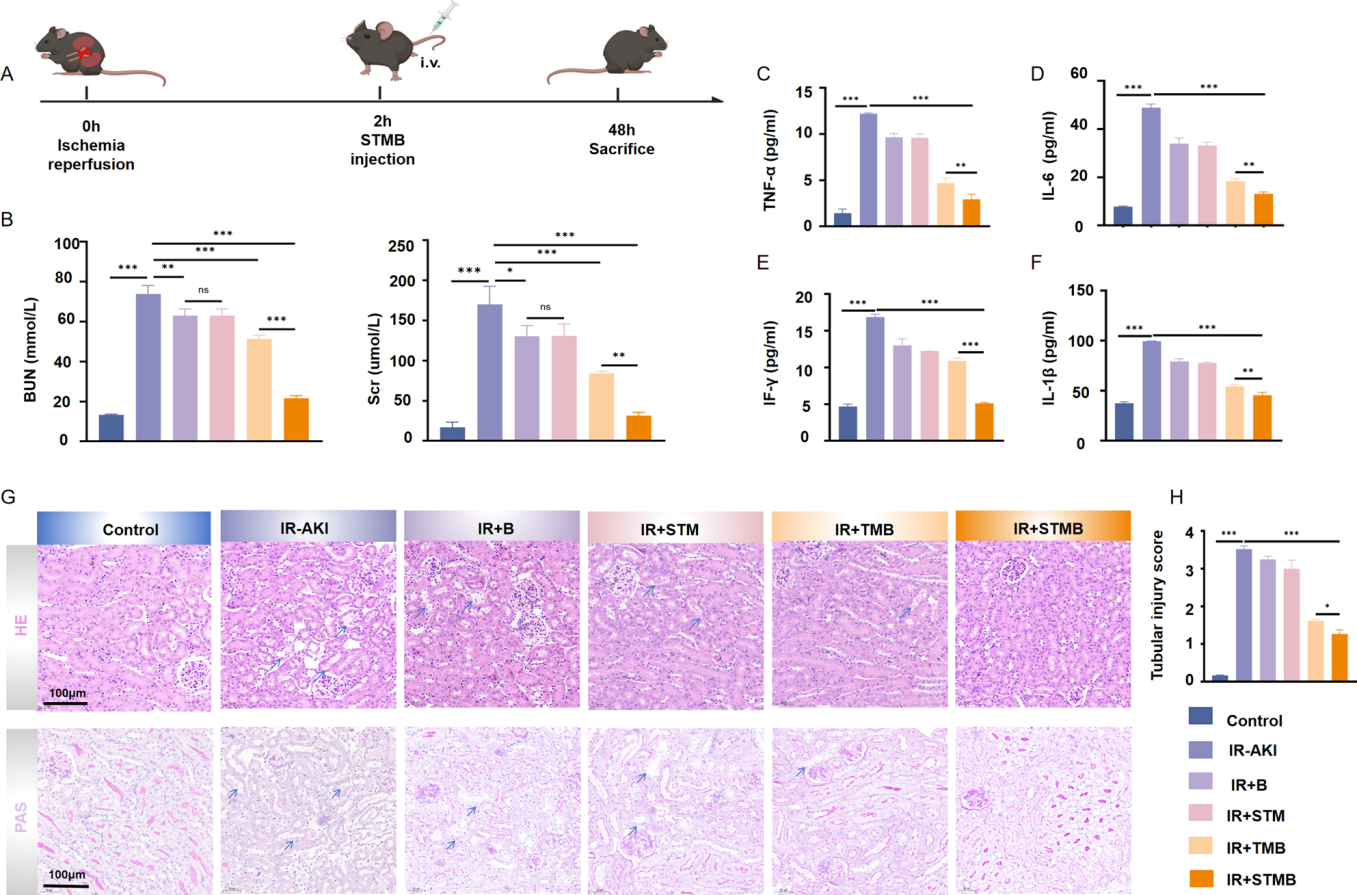
STMB reduces renal injury and inflammation in mouse models of I/R-mediated AKI. (**A**) Schematic illustration depicting I/R AKI mouse model establishment and subsequent therapeutic regimen. (**B**) Evaluation of mouse renal function (BUN, SCr) in I/R AKI mice after various treatments. *n* = 3. (**C**–**F**) Detection of inflammatory marker expression levels in mouse serum using ELISA. *n* = 5. (**G**) Representative images of I/R model renal tissue stained with H&E and PAS. Scale bar: 100 μm. Blue arrow represents brush-like edge of the proximal tubule and the exposed tubule basement membrane. (**H**) Quantitative analysis of tubular injury score in different treatment groups stained with H&E. *n* = 3, *p < 0.05; **p < 0.01; and ***p < 0.001

I/R-induced AKI is another prevalent experimental model that effectively mimics the pathophysiology of intrinsic AKI^28^. An IR-induced AKI mouse model was established to investigate the potential protective effect of STMB on IR-associated AKI, and STMB was administered intravenously for potential intervention (Fig. 7A). Serum biochemical analysis confirmed the successful establishment of the IR-induced AKI model, with I/R challenge triggering dramatic elevations in both blood BUN and SCr levels compared to those in the controls (*p* < 0.001). While all treatment groups demonstrated some degree of renal function improvement, the STMB injection produced the most significant reductions in these biomarker levels, approaching near-normal levels (*p* < 0.001 vs. IR-AKI; Fig. 7B). The inflammatory component of IR-AKI was evident through markedly elevated serum concentrations of pro-inflammatory cytokines. Quantitative analysis using ELISA showed that all treatment groups had decreased IL-6, TNF-α, IFN-γ, and IL-1β levels, with the most significant reduction noted in the STMB group (*p* < 0.001 vs. IR-AKI; Fig. 7C–F). The histopathological examination with H&E staining revealed extensive renal tubular damage in IR-treated mice, as manifested by the brush-like edge of the proximal tubule and the exposed tubule basement membrane (Fig. 7G). Notably, the STMB injection ameliorated these pathological alterations (*p* < 0.001 vs. IR-AKI; Fig. 7G–H). In conclusion, these results indicate that intravenous STMB can significantly improve IR-related AKI, with the same therapeutic effect as previously obtained in the Cis-induced AKI model.

### Biosafety assessment

**Fig. 8:**
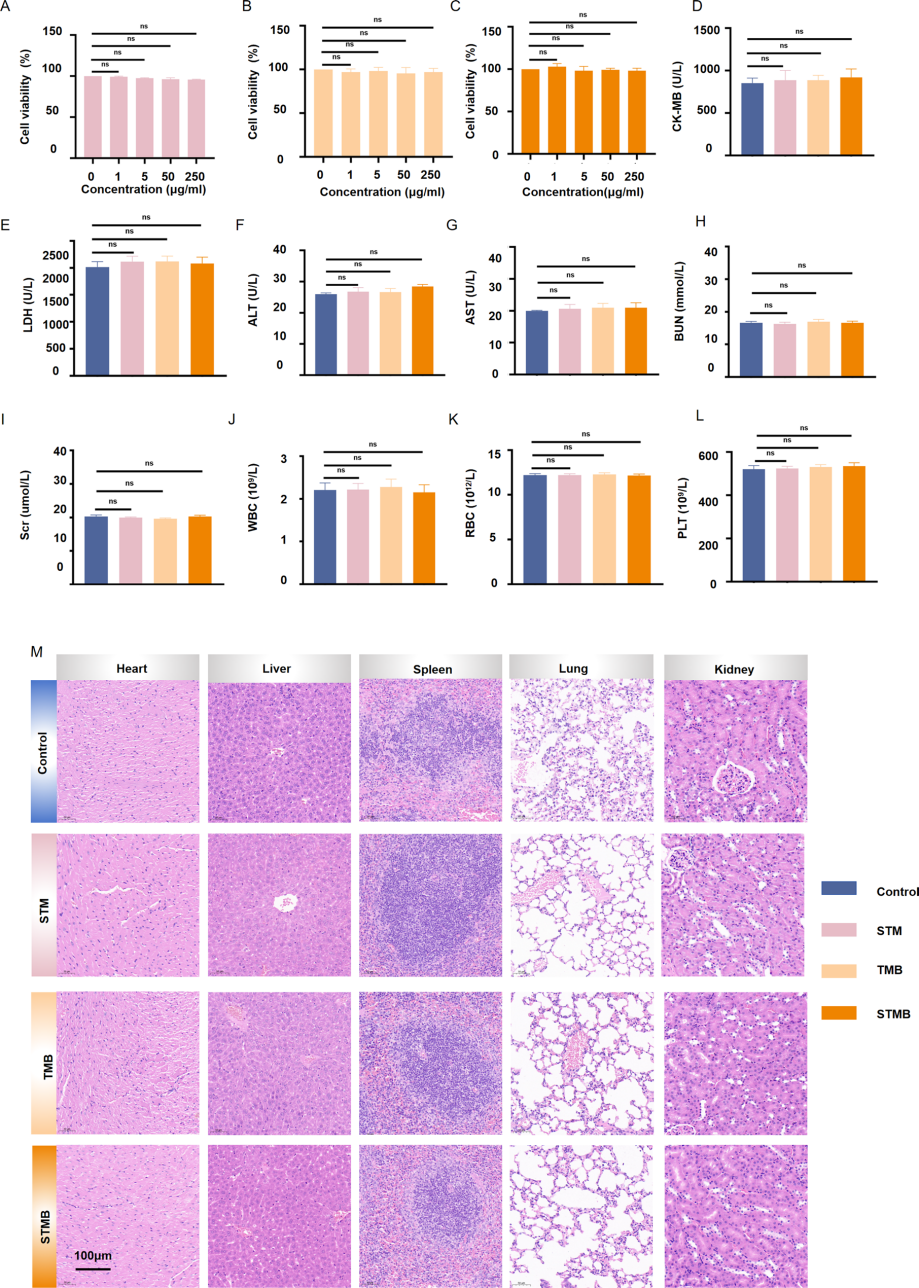
Biosafety STMB assessment in vitro and *in vivo.* HK-2 cell viability at different equivalent concentrations of (**A**) STM, (**B**) TMB, and (**C**) STMB for 24 h. (**D–I**) Blood samples were collected for cardiac, hepatic, and renal function tests. CK-MB, creatine kinase isozyme; LDH, lactate dehydrogenase; ALT, alanine transaminase; AST, aspartate transaminase; BUN, blood urea nitrogen; SCr, serum creatinine. (**J–L**) Blood samples were collected for hematocyte analysis. WBC, leukocyte; RBC, erythrocyte; PLT, platelet. (**M**) C57BL/6J mice were injected with STM, TMB, and STMB via the tail vein for 21 days and major organs were harvested for H&E staining. Scale bar: 100 μm. *n* = 5, *p < 0.05; **p < 0.01; and ***p < 0.001

Cell viability assays demonstrated no statistically significant differences in cell activity measurements among different concentrations of STM, TMB, and STMB when co-cultured with HK-2 cells for 24 h. Notably, high-concentration NPs (250 µg mL^− 1^) showed no detectable cytotoxicity when co-cultured with HK-2 cells, indicating excellent NP biocompatibility (Fig. 8A–C). A multiorgan toxicity assessment was carried out to comprehensively analyze the long-term biosafety of NPs (50 mg kg^− 1^, 5X the therapeutic dose) in vivo, evaluating critical systems (e.g., cardiovascular, hepatic, pulmonary, and splenic) in mice subjected to a 21-day high-dose treatment. To quantitatively corroborate the NP safety profile, serum samples were collected from mice and analyzed in terms of various biochemical parameters, including creatine kinase isozyme (CK-MB), lactate dehydrogenase (LDH), alanine aminotransferase (ALT), aspartate aminotransferase (AST), BUN, SCr, leukocyte (WBC), erythrocyte (RBC), and platelet (PLT) levels. The evaluated biochemical values were comparable among the four experimental groups, showing both normal baseline levels and an absence of significant intergroup differences (Fig. 8D–L). Collectively, these findings substantiate the favorable biocompatibility and safety of all tested NPs in vivo. Furthermore, the histological examination utilizing H&E staining revealed no evident tissue damage in any of the NP-treated groups compared to the control animals (Fig. 8M). This observation indicates the absence of discernible organ lesions following a prolonged injection.

## Discussion

The poor regenerative ability of RTECs in the face of chronic inflammatory stress remains a key factor perpetuating a cycle of injury, making AKI a persistent clinical concern [39–41]. The present study proposed a novel therapeutic paradigm by integrating mitochondrial dysfunction correction, oxidative stress resolution, and immune microenvironment remodeling via a spatiotemporally coordinated nanoplatform STMB. The study findings demonstrated that dual-targeting of mtDNA leakage and ROS overproduction halts injury progression and actively initiates RTECs regeneration, thereby addressing the core limitations of the current AKI therapies.

Recent studies emphasize that mtDNA leakage is a key driver of innate immune overactivation in AKI, likely due to the release of mtDNA from damaged mitochondria triggering the cGAS-STING pathway activation in RTECs [42] and leading to persistent NF-κB signaling and ultimately AKI [43]. However, there are currently no interventions directly targeting this process. This mechanism explains why conventional anti-inflammatory or antioxidant therapies often fail to restore tubular integrity. By contrast, STMB achieves synergistic blockade, where BAM15 stabilizes MMP to prevent mtDNA release at the source, while TA-Mn chelates scavenge ROS and trap residual mtDNA via π-π/H-bond interactions. This dual-action strategy breaks the cycle of inflammation-regeneration imbalance, which is a significant advance over single-target approaches.

The hierarchical targeting capability of STMB addresses a long-standing obstacle in renal drug delivery, which is the inability to achieve both organ-specific accumulation and subcellular precision. The Ser-modified shell exploits the upregulated KIM-1 receptor in injured RTECs for renal tubule enrichment, overcoming systemic NP dispersion observed in previous designs (e.g., PEGylated carriers) [44, 45]. At the same time, TA’s intrinsic mitochondrial affinity enables precise subcellular localization. Metal ions can confer enzymatic activity to nanomedicines [46–48]. In STMB, the manganese ions not only serve as a structural stabilizer but also impart to STMB a function similar to a reducing enzyme, which mediates the structural disintegration caused by ROS, thereby ensuring the precise release of STMB in mitochondria.This targeted logic significantly enhanced therapeutic efficacy while minimizing off-target effects, as evidenced by the reduction in mtDNA levels in the cytoplasm compared to those in the untreated control group.

Notably, STMB’s therapeutic effects extend beyond epithelial repair. The system promotes macrophage polarization toward M2 phenotype by reducing mtDNA-driven inflammation, which may further secrete pro-regenerative cytokines to support tubular reconstruction. The synergistic interplay between this oxidative repair and immunoremodeling highlights the importance of integrating RTECs and immunomodulatory interventions in AKI management, an aspect that has been overlooked by most previous studies focusing solely on RTECs.

While current results in Cis- and I/R-induced AKI models are promising, translational potential requires further validation. First, the long-term biosafety of Mn ion release kinetics needs systematic evaluation, although our preliminary data show no hepatotoxicity or cardiotoxicity within 21 days. Second, the heterogeneity of AKI etiologies (e.g., sepsis-associated AKI) may demand additional functional customization of the nanoplatform. Nevertheless, the present work establishes a proof-of-concept framework for multitiered AKI therapy, bridging the gap between molecular pathology and nanomedicine design. Building on the present study, three directions warrant exploration as follows: (1) incorporating real-time mtDNA leakage sensors into the nanocarrier for theranostic applications; (2) optimizing dosage regimens to balance mitochondrial uncoupling and energy supply during repair; and (3) extending this “injury-suppression/regeneration-activation” dual-mechanism to other I/R injury models. Such efforts may pave the way for personalized AKI therapeutics with spatiotemporal precision.

## Conclusion

In conclusion, STMB NPs were constructed utilizing a simple coordinated self-assembly approach and their role as a potent antioxidant defense platform was verified, showing significant efficacy in ameliorating AKI. This system exhibits targeted delivery to the kidney, RTECs, and mitochondria. STMB possesses the capability to effectively scavenge ROS, deploy multiple antioxidant mechanisms, mitigate inflammation, diminish mtDNA release, modulate macrophage polarization, reduce apoptosis, and provide protection against I/R injury as well as Cis-induced AKI, ultimately leading to the restoration of renal function. The notable antioxidant activity and high biocompatibility of STMB NPs underscore their promising applicability in the clinical management of AKI and other diseases characterized by aberrant ROS levels, positioning it as a novel and highly potent antioxidant nanomedicine.

## Experimental section

### Materials and reagents

BAM15 and Cy5 were procured from MedChemExpress (Shanghai, China). TA and Ser were sourced from Aladdin (Shanghai, China). The Cell Counting Kit-8 (CCK-8), enhanced chemiluminescence (ECL) reagent, 2′,7′-dichlorodihydrofluorescein (DCFH-DA), total antioxidant capacity ABTS, hydrogen peroxide (H₂O₂) assay kit, superoxide (O₂•−), hydroxyl free radical (·OH), calcium green-acetoxymethyl ester/propidium iodide (calcein-AM/PI), JC-1 mitochondrial superoxide, and mPTP were sourced from Beyotime Biotechnology (Shanghai, China). Annexin V-fluorescein isothiocyanate (FITC) and PI double-staining kit was obtained from Abcam (Cambridge, UK). The TUNEL Apoptosis Assay Kit was obtained from Elabscience (Wuhan, China).

Antibodies against cleaved caspase-3 (ab214430), Bcl-2-associated X protein (ab182733), B-cell lymphoma2 (ab194583), and KIM-1 (NBP1-76701) were from Novus (Centennial, CO, USA). NLRP3 (A5652) and TLR-9 (A14642) were acquired from ABclonal (Wuhan, China). Alexa Fluor™ 488-conjugated anti-rabbit IgG antibody (ab150077) and Alexa Fluor™ 647-conjugated anti-rabbit IgG antibody (ab150079) were also from Abcam (Cambridge, UK). To evaluate the levels of pro-inflammatory mediators, such as IL-6, TNF-α, IFN-γ, and IL-1β, corresponding ELISA kits were procured from Bio-Techne (USA).

### TMB and STMB synthesis

Mesoporous silica nanoparticles (MSNs, 10-nm pore size; Xi’an Ruixi Biotechnology Co., Ltd) were pre-dried under vacuum at 60 °C for 2 h. BAM15 (4 mg) dissolved in anhydrous DMSO (1 mL) was added to MSNs (80 mg) and the mixture was stirred under reduced pressure (0.1 MPa, 300 × *g*) for 12 h at 25 °C to facilitate drug infiltration. The sample was then centrifuged (12,000 × *g*, 10 min) and the resulting solid material was dispersed in deionized water (80 mL) followed by the addition of TA (40 mg) and MnCl₂·4 H₂O (15 mg) under sonication. The solution pH was subsequently titrated to 7.4 using 0.1 M NaOH and the sample was incubated under continuous stirring at room temperature for 3 h. The resulting precipitate was isolated via centrifugation (12,000 × *g*, 10 min), rinsed three times with a water/ethanol mixture, and incubated with Na₂CO₃ (200 mg, 50 °C, 12 h) to remove MSNs. For STMB synthesis, Ser (2.25 mg) was conjugated to the surface of TMB NPs via hydrogen bonding and metal coordination while stirring (300 × *g*, 12 h). The drug loading capacity (DLC) and drug-loading efficiency (DLE) of BAM15 were calculated using the following formulas:

$$\text{DLC } (\%) = \mathrm{M}_{\mathrm{drug}}/\mathrm{M}_{\mathrm{nanoparticle}}\times 100\% (\text{M: Mass}).$$

$$\text{DLE }(\%) = \mathrm{DLC}_{\mathrm{measured}}/\mathrm{DLC}_{\mathrm{theoretical}}\times 100\%.$$

### Antioxidant activity and ROS scavenging capacity of STMB

#### Measurement of free radical scavenging capacity

The ABTS free radical scavenging assay was employed to evaluate the radical scavenging ability of STMB according to the kit’s standard protocols. Specifically, ABTS and peroxidase working solutions were prepared and pre-mixed as recommended by the manufacturer. STMB was introduced at varying concentrations (0.1, 0.5, 1, 5, 10, and 50 µg mL^− 1^) into the reaction mixture. Following a 6-min incubation at room temperature, the absorbance was recorded at 405 nm with a multifunctional microplate reader (Tecan Spark). Each assay was carried out with five replicates.

#### Measurement of ·OH scavenging capacity

The ·OH scavenging activity of STMB was evaluated across a gradient of concentrations (as described in Measurement of free radical scavenging capacity). It was carried out with a commercially available assay kit following the supplier’s recommended procedures. The ·OH is generated via a Fenton reaction between H₂O₂ and Fe²⁺. These radicals oxidize Fe²⁺ to Fe³⁺ in the phenanthroline-Fe²⁺ system, leading to a reduction in absorbance at 536 nm. The ability of STMB to scavenge hydroxyl radicals is indicated by its capacity to inhibit this decrease in absorbance. The reaction reagent was mixed thoroughly with STMB. After incubation at 37 °C for 30 min, the optical density values of the blank, control, and test solutions were recorded at 536 nm. Each assay was carried out with five replicates.

#### Measurement of H_2_O_2_ scavenging

A catalase (CAT) assay kit was used to determine the ability of STMB to scavenge H_2_O_2_. H₂O₂ exhibits a characteristic absorption peak at 240 nm. CAT catalyzes the decomposition of H₂O₂ into water and oxygen, leading to a reduction in absorbance at this wavelength. CAT activity can be quantified based on the rate of decrease in absorbance over time. Varying concentrations of STMB (same as those in Sect. 5.3.1) were added to the reaction system according to the kit instructions. Absorbance was recorded at 240 nm. Each assay was carried out with five replicates.

#### Measurement of O_2_^•−^ scavenging capacity

The O_2_^•−^ scavenging capacity of STMB at varying concentrations (same as those in Sect. 5.3.1) was evaluated using a O_2_^•−^activity content assay kit according to the manufacturer instructions. The O₂•⁻ converts hydroxylamine hydrochloride to nitrite (NO₂⁻), which subsequently reacts with sulfanilic acid and N-(1-naphthyl)ethylenediamine dihydrochloride to form a red azo dye. The resulting compound exhibits a distinct absorbance maximum at 530 nm upon formation. The reaction mixture was subsequently incubated at 37 °C for 10 min, followed by measurement of the blank and test solution absorbance values at the specified wavelength. Each assay was carried out with five replicates.

### Hemolysis test

A hemolysis assay was carried out to assess the potential hematological toxicity of STMB. Fresh blood was collected from Sprague-Dawley rats and centrifuged at 1,000 × *g* for 5 min. After removing plasma and buffy coat, the remaining RBCs were repeatedly rinsed with phosphate-buffered saline (PBS) until the supernatant appeared transparent. The washed RBCs were then reconstituted as a 2% (v/v) suspension in PBS. Subsequently, 1 mL of each NP formulation (STMB dispersed in PBS at concentrations ranging from 0.1 to 50 µg mL^− 1^) was combined with an equal volume of the RBC suspension. Control samples consisted of RBCs mixed with PBS (representing 0% hemolysis) and ultrapure water (representing 100% hemolysis). All mixtures were incubated for 3 h at 37 °C with continuous shaking at 100 × *g*. After the incubation, the samples were centrifuged again at 1,000 × *g* for 5 min to pellet any intact RBCs. The supernatant from each sample was analyzed for absorbance at 541 nm. The proportion of hemolysis was determined using the equation below:

$$\begin{aligned}\text{Hemolysis }(\%)&=(\mathrm{Ab}_{\mathrm{sampel}}-\mathrm{Ab}_{\text{negative control}})/\\&\quad (\mathrm{Ab}_{\text{positive control}}-\mathrm{Ab}_{\text{negative control}}) \\ &\quad\times 100\%.(\text{Ab: Absorbance}).\end{aligned}$$

### Cell culture and treatments

The HK-2 human renal proximal tubular cell line was procured from the American Type Culture Collection (ATCC, USA) and the Chinese Academy of Sciences (Shanghai, China). Cells were cultured in DMEM enriched with 10% FBS (Moregate, Australia) and 1% penicillin-streptomycin (Invitrogen, USA) at 37 °C in a 5% CO₂ humidified incubator. For AKI modeling, the cells were treated with 20 mM Cis (Sigma-Aldrich) for 24 h. Successful establishment of the Cis-induced AKI model was verified through microscopic observation of typical injury-related morphological alterations, such as cell rounding, shrinkage, and detachment, coupled with a marked decline in viability relative to that in untreated groups measured via the CCK-8 assay.

### ROS detection

Intracellular ROS levels were measured using an ROS Assay Kit (Beyotime, China). Cells were exposed to 20 mM Cis (Sigma-Aldrich) or left untreated, followed by incubation with 10 µM 2′,7′-dichlorofluorescin diacetate (DCFH-DA) for 20 min at 37 °C, washed, and analyzed by flow cytometry (CytoFLEX, Beckman Coulter, USA). Three independent experiments were performed.

### MMP assessment

MMP was evaluated using JC-1 fluorescent dye (Beyotime, China). HK-2 cells were labeled with JC-1 dye, followed by incubation, washing steps, and subsequent flow cytometric analysis (CytoFLEX, Beckman Coulter, USA). MMP was observed using laser confocal microscopy (FV4000, Olympus, Japan).

### Apoptosis analysis by flow cytometry

Apoptosis in response to Cis exposure was assessed using flow cytometry with FITC and PI double staining following the recommended manufacturer procedure. HK-2 cells were plated in 6-cm dishes at a density of 8 × 10⁵ cells per dish and exposed to Cis for 24 h. After 2 h of the Cis treatment, BAM15, STM, TMB, or STMB was added to the culture dish and stained with Annexin V-FITC/PI solution. After incubation, the cells were washed and analyzed using flow cytometry. Three independent experiments were conducted.

### Establishment of Cis-induced AKI model

Male C57BL/6 mice (6–8 weeks old, weighing 18–22 g) were acquired from Hunan SJA Laboratory Animal Co., Ltd (Changsha, China) and housed under specific pathogen-free conditions. The animals were utilized to generate a Cis-induced AKI model for subsequent experiments. The mice received a single intraperitoneal Cis injection (20 mg kg^− 1^ in normal saline; Sigma-Aldrich, St. Louis, MO, USA) and were monitored for 48 h before sacrifice. Kidney tissue and blood samples were collected for further analysis.

### Tubular targeting of STMB investigation

Cy5-STMB (10 mg kg^− 1^) was administered via tail vein for 12 h in AKI mice. The kidneys were excised, sectioned, and stained with(marker for proximal renal tubules FITC-labeled Lotus tetragonolobus lectin (Abcam, Cambridge, UK) for 1 h. Nuclear cells were immunolabeled with 4′,6-diamidino-2-phenylindole (DAPI). High-resolution images capturing STMB distribution were acquired with an Olympus FV4000 (Japan) laser confocal microscope.

### Serum biochemistry

Serum was separated from blood samples by centrifugation (11,000 × g, 15 min) for subsequent quantification of BUN and SCr with designated assay kits (Nanjing Jiancheng Bioengineering Institute), following the vendor’s specified protocol.

### Ischemic AKI model

Bilateral ischemia was induced by clamping both renal pedicles for 22 min followed by reperfusion for 48 h. Body temperature was maintained at 36.6–36.8℃ throughout the procedure. Mice were sacrificed upon experiment completion, kidney tissue was fixed in 4% paraformaldehyde, and serum samples were collected, stored at −80 °C, and reserved for further biochemical assays.

### Kidney histopathological analysis

The kidneys were fixed with 4% polyformaldehyde, washed with gradient ethanol concentrations, paraffin-embedded, and processed into histological slices. Standard H&E and PAS staining methods were utilized, followed by graded alcohol dehydration, xylene clearing, and mounting. Light microscopy was employed for morphological examination of kidney tissues, with images captured during the process.

### Microstructure observation in mitochondria

The renal samples were initially fixed in 2.5% glutaraldehyde solution overnight, followed by rinsing in PBS and subsequent post-fixation with 1% osmium tetroxide. The samples were infiltrated and embedded in epoxy resin after dehydration in a graded ethanol series. Ultrathin Sects. (200–400 nm) were mounted on nickel grids, stained with uranyl acetate and lead citrate, and imaged using TEM (FEI Tecnai F20, USA).

### Immunohistochemical assessment of macrophage infiltration

The number of macrophages in renal tissues and their inflammatory levels were evaluated. Kidney tissue sections, after de-waxing and rehydration, were treated with blocking buffer for 30 min at room temperature. They were then incubated successively with a primary anti-F4/80 antibody (Servicebio, GB113373-100, China) and an HRP-conjugated goat anti-rabbit IgG secondary antibody (Servicebio, GB23303, China). Light microscopy images were obtained, where brown-stained cells were identified as positive cells.

### High-throughput transcriptome sequencing and bioinformatics processing

Total RNA extraction from renal tissues of both AKI and control mice was conducted utilizing TRIzol Reagent (Invitrogen, China).All procedures were performed in strict adherence to the manufacturer’s guidelines. The RNA integrity, purity, and concentration were verified by Wuhan Seqhealth Technology Co., Ltd. During data preprocessing, adapter-contaminated sequences, reads comprising over 5% ambiguous bases (N), and low-quality reads with > 20% low-confidence bases were filtered out to yield clean reads for downstream analysis. Sequencing was carried out on the Illumina HiSeq X Ten platform (California, USA). Gene expression profiling was conducted to identify differentially expressed genes by comparing AKI and normal groups, with genes meeting the criteria of |log₂FoldChange| > 1 and adjusted p-value of < 0.01 classified as differentially expressed genes. Pathway enrichment analysis was conducted using the Student’s t-test with multiple testing corrections, retaining pathways that met a false discovery rate threshold of 5%.

### Immunofluorescence detection of MtDNA release

HK-2 cells were pretreated with STMB (1 µg mL^− 1^) for 2 h with or without Cis (20 µM) for 24 h. Live cells were stained with MitoTracker Deep Red (200 nM; Beyotime, C1035) for 30 min. Following a 15-minute fixation step using 4% paraformaldehyde and subsequent permeabilization with 0.3% Triton X-100 for 10 min, immunostaining was performed using anti-dsDNA antibody (Abcam, ab27156) at 4 °C overnight, followed by incubation with Alexa Fluor 488-conjugated secondary antibody (1:500; Beyotime, A4028) for 1 h. Nuclei were counterstained with DAPI. Confocal images were analyzed in ImageJ(NIH, USA), where the dsDNA signal intensity in cytoplasmic mtDNA was quantified as the MitoTracker-negative/DAPI-negative area ratio.

### TUNEL detection in renal tissue

Protease K was used to remove DNA-binding proteins after fixing the renal tissue samples with 4% polyformaldehyde. The TUNEL assay (Elabscience, China) was carried out following the supplier’s recommended protocol to evaluate apoptotic cells. Apoptotic renal cells (green fluorescence) were counted under a confocal microscope (Leica SP8, German). Ten random fields per specimen were analyzed at 400x magnification, and the percentage of TUNEL-positive cells was recorded.

### WB analysis

WB was conducted by collecting kidney samples, which were then lysed using radio immunoprecipitation assay buffer (Beyotime, China) supplemented with 1% phenylmethanesulfonyl fluoride (Beyotime, China). Following centrifugation at 12,000 × *g* for 15 min at 4 °C to pellet insoluble debris, the protein concentration was quantified with a bicinchoninic acid assay kit (Thermo Fisher Scientific). Subsequently, total protein samples were separated by electrophoresis on 12.5% SDS-polyacrylamide gels. Proteins were transferred to polyvinylidene fluoride membranes following electrophoresis, which were then blocked with 5% bovine serum albumin buffer. Membranes were subsequently incubated overnight at 4℃ with primary antibodies against KIM-1, BAX, Bcl2, cleaved caspase-3, NLRP3, and TLR9. Following three washes with TBST, the membranes were incubated with an HRP-linked goat anti-rabbit IgG secondary antibody (Servicebio, GB23303). ECL WB detection reagents were then applied.

### Inflammatory cytokine gene detection

The concentrations of key inflammatory cytokine genes, including TNF-α, IL-6, IFN-γ, and IL-1β, were determined using commercially available ELISA kits according to the manufacturer protocols. Five independent experiments were performed (*n* = 5).

### Cytotoxicity *in vitro*

HK-2 human renal tubular epithelial cells were seeded into 96-well plates at an initial density of 5 × 10³ cells per well. Cells were maintained in DMEM supplemented with 10% FBS and 1% penicillin–streptomycin, and incubated at 37 °C in a 5% CO₂ humidified atmosphere for 24 h. Then, the cells were treated with different concentrations of STM, TMB, or STMB for an additional 24 h under the same conditions. Cell viability and metabolic activity were assessed with the CCK-8 assay.

### Assessment of toxicity *in vivo*

C57BL/6J mice received daily intravenous injections of STM, TMB, or STMB (50 mg kg^− 1^) through the tail vein over a period of 21 days. Upon completion of the treatment, major organs, including the heart, liver, spleen, lung, and kidney, were collected and fixed with 4% paraformaldehyde (Sigma-Aldrich, USA). The samples were then processed through paraffin embedding, thin-sectioning, and H&E staining (Beyotime Biotechnology, China) for subsequent histopathological examination. Blood samples were obtained from treated mice for comprehensive biochemical and hematological evaluation. Cardiac function was determined by quantifying CK-MB and LDH levels. Liver function was assessed via measurements of ALT and AST levels, while kidney function was monitored through blood BUN and SCr assays. Hematologic profiles encompassing WBC, RBC, and PLT counts were examined to evaluate the potential impact on hematopoietic parameters. All biochemical tests were carried out using commercially supplied kits from Nanjing Jiancheng Bioengineering Institute (China). Complete blood counts were acquired using an automated hematology analyzer (Mindray, China).

### Statistical analysis

Data were expressed as mean ± standard deviation. Comparisons used one-way ANOVA with Tukey’s post-hoc test (GraphPad Prism 9.0, GraphPad Software, USA), where significance was indicated by *p < 0.05, **p < 0.01, and ***p < 0.001.

## Supplementary Information

Supplementary Material 1.

## Data Availability

No datasets were generated or analysed during the current study.

## Declarations

All animal procedures were approved by the Chongqing Medical University Animal Care and Use Committee (approval no.: IACUC-SAHCQMU-2023-0061).

## References

[1] Su L, Zhang J, Gomez H, Kellum JA, Peng Z. Mitochondria ROS and mitophagy in acute kidney injury. Autophagy. 2023;19:401–14. 35678504 10.1080/15548627.2022.2084862 PMC9851232

[2] Qi Y, Zheng J, Zi Y, Song W, Chen X, Cao S, et al. Loureirin C improves mitochondrial function by promoting NRF2 nuclear translocation to attenuate oxidative damage caused by renal ischemia-reperfusion injury. Int Immunopharmacol. 2024;138:112596. 38981224 10.1016/j.intimp.2024.112596

[3] Kwiatkowska E, Kwiatkowski S, Dziedziejko V, Tomasiewicz I, Domański L. Renal microcirculation injury as the main cause of ischemic acute kidney injury development. Biology (Basel). 2023. 10.3390/biology12020327. 36829602 10.3390/biology12020327 PMC9953191

[4] Matsuura R, Doi K, Rabb H. Acute kidney injury and distant organ dysfunction–network system analysis. Kidney Int. 2023;103:1041–55. 37030663 10.1016/j.kint.2023.03.025

[5] Zhang C, Xiang Z, Yang P, Zhang L, Deng JLiao XA. Nano-immunomodulatory systems for the treatment of acute kidney injury.Advanced science, vol. 12. Baden-Wurttemberg, Germany: Weinheim; 2025. p. e2409190. 10.1002/advs.202409190 PMC12061249 40145715

[6] Yang Y, Jin S, Zhang J, Chen W, Lu Y, Chen J, et al. Acid-sensing ion channel 1a exacerbates renal ischemia-reperfusion injury through the NF-κB/NLRP3 inflammasome pathway. J Mol Med. 2023;101:877–90. 37246982 10.1007/s00109-023-02330-7 PMC10300185

[7] Neyra JA, Ortiz-Soriano V, Liu LJ, Smith TD, Li X, Xie D, et al. Prediction of mortality and major adverse kidney events in critically ill patients with acute kidney injury. Am J Kidney Dis. 2023;81:36–47. 35868537 10.1053/j.ajkd.2022.06.004 PMC9780161

[8] Nie Y, Wang L, Liu S, Dai C, Cui T, Lei Y, et al. Natural ursolic acid based self-therapeutic polymer as nanocarrier to deliver natural resveratrol for natural therapy of acute kidney injury. J Nanobiotechnology. 2023;21:484. 38105186 10.1186/s12951-023-02254-x PMC10726514

[9] Chen Q, Nan Y, Yang Y, Xiao Z, Liu M, Huang J, et al. Nanodrugs alleviate acute kidney injury: manipulate RONS at kidney. Bioact Mater. 2023;22:141–67. 36203963 10.1016/j.bioactmat.2022.09.021 PMC9526023

[10] Wang X, Luo T, Yang Y, Yang L, Liu M, Zou Q, Wang D, Yang C, Xue Q, Liu S, Wan J, He G, Zeng A, Hou J, Ma S, Wang P. TRPA1 protects against contrast-induced renal tubular injury by preserving mitochondrial dynamics via the AMPK/DRP1 pathway. Free radical biology & medicine. 2024;224:521–39. 39278575 10.1016/j.freeradbiomed.2024.09.012

[11] Liu YT, Zhang H, Duan SB, Wang JW, Chen H, Zhan M, et al. Mitofusin2 ameliorated endoplasmic reticulum stress and mitochondrial reactive oxygen species through maintaining mitochondria-associated endoplasmic reticulum membrane integrity in Cisplatin-induced acute kidney injury. Antioxid Redox Signal. 2024;40:16–39. 37053105 10.1089/ars.2022.0178

[12] Shao B, Wang HD, Ren SH, Chen Q, Wang ZB, Xu YN, et al. Exosomes derived from a mesenchymal-like endometrial regenerative cells ameliorate renal ischemia reperfusion injury through delivery of CD73. Stem Cell Res Ther. 2025;16:148. 40140882 10.1186/s13287-025-04275-9 PMC11948919

[13] Yu H, Li Q, Zhu H, Liu C, Chen W, Sun L. Mesenchymal stem cells attenuate systemic lupus erythematosus by inhibiting NLRP3 inflammasome activation through Pim-1 kinase. Int Immunopharmacol. 2024;126:111256. 37992447 10.1016/j.intimp.2023.111256

[14] Yang J, Yuan L, Li L, Liu F, Liu J, Chen Y, et al. Trehalose activates autophagy to alleviate cisplatin-induced chronic kidney injury by targeting the mTOR-dependent TFEB signaling pathway. Theranostics. 2025;15:2544–63. 39990216 10.7150/thno.102559 PMC11840734

[15] Bui AP, Pham TTM, Kim M, Park JH, Kim JI, Seo JH, et al. GLDC alleviates cisplatin-induced apoptosis, cellular senescence, and production of reactive oxygen species via regulating UCP1 in the kidney. Life Sci. 2025;368:123502. 40010632 10.1016/j.lfs.2025.123502

[16] Kim J, Kim HS, Chung JH. Molecular mechanisms of mitochondrial DNA release and activation of the cGAS-STING pathway. Exp Mol Med. 2023;55:510–9. 36964253 10.1038/s12276-023-00965-7 PMC10037406

[17] Zecchini V, Paupe V, Herranz-Montoya I, Janssen J, Wortel IMN, Morris JL, Ferguson A, Chowdury SR, Segarra-Mondejar M, Costa ASH, Pereira GC, Tronci L, Young T, Nikitopoulou E, Yang M, Bihary D, Caicci F, Nagashima S, Speed A, Bokea K, Baig Z, Samarajiwa S, Tran M, Mitchell T, Johnson M, Prudent J, Frezza C. Fumarate induces vesicular release of MtDNA to drive innate immunity. Nature. 2023;615:499–506. 36890229 10.1038/s41586-023-05770-w PMC10017517

[18] Huang Y, Ning X, Ahrari S, Cai Q, Rajora N, Saxena R, et al. Physiological principles underlying the kidney targeting of renal nanomedicines. Nat Rev Nephrol. 2024;20:354–70. 38409369 10.1038/s41581-024-00819-z PMC12875306

[19] Yu P, Gu T, Rao Y, Liang W, Zhang X, Jiang H, et al. A novel marine-derived anti-acute kidney injury agent targeting peroxiredoxin 1 and its nanodelivery strategy based on ADME optimization. Acta Pharm Sin B. 2024;14:3232–50. 39027260 10.1016/j.apsb.2024.03.005 PMC11252462

[20] Zhang J, Gao B, Ye B, Sun Z, Qian Z, Yu L, Bi Y, Ma L, Ding Y, Du Y, Wang W, Mao Z. Mitochondrial-Targeted Delivery of Polyphenol-Mediated Antioxidases Complexes against Pyroptosis and Inflammatory Diseases. Advanced materials (Deerfield Beach, Fla.). 2023;35:e2208571. 10.1002/adma.202208571 36648306

[21] Li Z, Feng Y, Zhang S, Li T, Li H, Wang D, et al. A multifunctional nanoparticle mitigating cytokine storm by scavenging multiple inflammatory mediators of sepsis. ACS Nano. 2023;17:8551–63. 37129445 10.1021/acsnano.3c00906

[22] Tsuji N, Tsuji T, Yamashita T, Hayase N, Hu X, Yuen PS, et al. BAM15 treats mouse sepsis and kidney injury, linking mortality, mitochondrial DNA, tubule damage, and neutrophils. J Clin Invest. 2023. 10.1172/JCI152401. 36757801 10.1172/JCI152401 PMC10065071

[23] Li J, Duan Q, Wei X, Wu J, Yang Q. Kidney-targeted nanoparticles loaded with the natural antioxidant rosmarinic acid for acute kidney injury treatment. Small. 2022. 10.1002/smll.202204388. 36253133 10.1002/smll.202204388

[24] Chao D, Dong Q, Yu Z, Qi D, Li M, Xu L, Liu L, Fang Y, Dong SA-O (2022) Specific nanodrug for diabetic chronic wounds based on Antioxidase-Mimicking MOF-818 nanozymes. Journal of the American Chemical society. 144(51):23438–23447. 36512736 10.1021/jacs.2c09663

[25] Zheng Y, Yi H, Zhan Z, Xue SS, Tang G, Yu X, et al. Reactive oxygen/nitrogen species scavenging and inflammatory regulation by renal-targeted bio-inspired rhodium nanozymes for acute kidney injury theranostics. J Colloid Interface Sci. 2024;662:413–25. 38359505 10.1016/j.jcis.2024.02.054

[26] Zhao L, Yue Z, Wang G, Qin J, Ma H, Tang D, et al. Smilax glabra roxb. alleviates cisplatin-induced acute kidney injury in mice by activating the Nrf2/HO-1 signalling pathway. Phytomedicine. 2025;139:156550. 40043544 10.1016/j.phymed.2025.156550

[27] Kolbrink B, von Samson-Himmelstjerna FA, Murphy JM, Krautwald S. Role of necroptosis in kidney health and disease. Nat Rev Nephrol. 2023;19:300–14. 36596919 10.1038/s41581-022-00658-w

[28] Yang C, Xu H, Yang D, Xie Y, Xiong M, Fan Y, et al. A renal YY1-KIM1-DR5 axis regulates the progression of acute kidney injury. Nat Commun. 2023. 10.1038/s41467-023-40036-z. 37460623 10.1038/s41467-023-40036-z PMC10352345

[29] Qi J, Luo Q, Zhang Q, Wu M, Zhang L, Qin L. Xue QNie X.Yi-Shen-Xie-Zhuo formula alleviates cisplatin-induced AKI by regulating inflammation and apoptosis via the cGAS/STING pathway. J Ethnopharmacol. 2023;309:116327. 36889420 10.1016/j.jep.2023.116327

[30] Zhang T, Widdop R, ERicardo. S D.Transition from acute kidney injury to chronic kidney disease: mechanisms, models, and biomarkers.American journal of physiology. Ren Physiol. 2024;327:F788–805. 10.1152/ajprenal.00184.2024 39298548

[31] Fan W, Wang C, Xu K. Liang HChi Q.Ccl5(+) macrophages drive pro-inflammatory responses and neutrophil recruitment in sepsis-associated acute kidney injury. Int Immunopharmacol. 2024;143:113339. 39418726 10.1016/j.intimp.2024.113339

[32] Yan J, Zhang J, Wang Y, Liu H, Sun X, Li A, et al. Rapidly inhibiting the inflammatory cytokine storms and restoring cellular homeostasis to alleviate sepsis by blocking pyroptosis and mitochondrial apoptosis pathways. Adv Sci. 2023;10:e2207448. 10.1002/advs.202207448 PMC10190643 36932048

[33] Zhu X, Wang X, Liu Z, Jiang B, He Z, Liu S, et al. Peroxidase-like nanozyme activates the cGAS‐STING pathway via ROS‐induced mtDNA release for cancer immunotherapy. Adv Funct Mater. 2024. 10.1002/adfm.202401576.40895409

[34] Lu P, Zheng H, Meng H, Liu C, Duan L, Zhang J, Zhang Z, Gao J. Zhang YSun T.Mitochondrial DNA induces nucleus pulposus cell pyroptosis via the TLR9-NF-κB-NLRP3 axis. J Translational Med. 2023;21:389. 10.1186/s12967-023-04266-5 PMC10273761 37322517

[35] Yao J, Sterling K, Wang Z, Zhang Y, Song W. The role of inflammasomes in human diseases and their potential as therapeutic targets. Signal Transduct Target Ther. 2024;9:10. 38177104 10.1038/s41392-023-01687-y PMC10766654

[36] Zuo S, Wang Y, Bao H, Zhang Z, Yang N, Jia M, Zhang Q, Jian A, Ji R, Zhang L, Lu Y. Huang YShen P.Lipid synthesis, triggered by PPARγ T166 dephosphorylation, sustains reparative function of macrophages during tissue repair. Nat Commun. 2024;15:7269. 39179603 10.1038/s41467-024-51736-5 PMC11343878

[37] Zhang NX, Guan C, Li CY, Xu LY, Xin YL, Song Z, Li TY, Yang CY, Zhao L, Che L, Wang YF. Man X FXu Y.Formononetin alleviates ischemic acute kidney injury by regulating macrophage polarization through KLF6/STAT3 pathway. Am J Chin Med. 2024;52:1487–505. 39169449 10.1142/S0192415X24500587

[38] Wang Y, Li C, Chen J, Cui X, Wang B, Wang Y, Wang D, Liu JLi. J.Pyxinol fatty acid ester derivatives J16 against AKI by selectively promoting M1 transition to M2c macrophages. J Agric Food Chem. 2024;72:7074–88. 38525502 10.1021/acs.jafc.3c06979

[39] Aggarwal S, Wang Z, Rincon Fernandez Pacheco D, Rinaldi A, Rajewski A, Callemeyn J, et al. SOX9 switch links regeneration to fibrosis at the single-cell level in mammalian kidneys. Science (New York, NY). 2024;383:eadd6371. 38386758 10.1126/science.add6371 PMC11345873

[40] Chen YT, Pan HC, Hsu CK, Sun CY, Chen CY, Chen YH, et al. Performance of urinary C-C motif chemokine ligand 14 for the prediction of persistent acute kidney injury: a systematic review and meta-analysis. Critical care. 2023;27:318. 37596698 10.1186/s13054-023-04610-7 PMC10439656

[41] Teixeira JP, Mayer KP, Griffin BR, George N, Jenkins N, Pal CA. González-Seguel FNeyra J A.Intensive care Unit-Acquired weakness in patients with acute kidney injury: A contemporary review. Am J Kidney Diseases: Official J Natl Kidney Foundation. 2023;81:336–51. 10.1053/j.ajkd.2022.08.028 PMC9974577 36332719

[42] Li J, Sun X, Yang N, Ni J, Xie H, Guo H, Wang X, Zhou L, Liu J, Chen S, Wang X, Zhang Y, Yu C. Zhang WLu L.Phosphoglycerate mutase 5 initiates inflammation in acute kidney injury by triggering mitochondrial DNA release by dephosphorylating the pro-apoptotic protein Bax. Kidney Int. 2023;103:115–33. 36089186 10.1016/j.kint.2022.08.022

[43] Diao L, Ma Y, Wang L, Li P, Zhang B, Meng W, Cai J, Meng Y, Zhou Y. Zhai JChen H.New insights into melatonin’s function on Thiacloprid-Induced pyroptosis and inflammatory response in head kidney lymphocytes of Cyprinus carpio: implicating mitochondrial metabolic imbalance and mtROS/cGAS-STING/NF-κB. Axis J Agricultural Food Chem. 2025;73:10574–88. 10.1021/acs.jafc.5c01019 40238706

[44] Jiang P, Kim W-SYT. Effective dispersion of CuPd alloy nanoparticles using the Taylor vortex flow for the preparation of catalysts with relatively clean surfaces. ACS Appl Nano Mater. 2022;5:9604–14.

[45] Schiffmann R, Goker-Alpan O, Vockley J, Wilcox WR, Ortiz D, Nie M, Shen J, Tavakkoli F. Kirn DFishman R.Cardiac effects of 4D-310 in adults with Fabry disease in a phase 1/2 clinical trial: Functional, quality of life, and imaging endpoints in patients with 12 months of follow up. Mol Genet Metab. 2023;138:107306.

[46] Hu Z, Shan J, Jin X, Sun W, Cheng L. Chen X-LWang X.Nanoarchitectonics of in situ Antibiotic-Releasing acicular nanozymes for targeting and inducing Cuproptosis-like death to eliminate Drug-Resistant bacteria. ACS Nano. 2024;18:24327–49. 39169538 10.1021/acsnano.4c06565

[47] Zhang S, Zhao X, Zhang W, Wei X, Chen X. -LWang X.Zn-DHM nanozymes regulate metabolic and immune homeostasis for early diabetic wound therapy. Bioactive Mater. 2025;49:63–84. 10.1016/j.bioactmat.2025.02.041 PMC11928983 40124598

[48] Zhao X, Zhang S, Wang M, Li Q, Wei X, Chen X. -LWang X.Cu-DHM nanozymes treat flap ischemia-reperfusion injury by amplifying immune modulation in a cascade manner and inhibiting cell apoptosis. Bioactive Mater. 2025;51:720–39. 10.1016/j.bioactmat.2025.06.036 PMC12242327 40641838

