# Supplementary material for: Renal-tubular-mitochondrial sequentially targeted nanoagent breaks the vicious cycle of oxidative stress and mtDNA-driven inflammation in acute kidney injury therapy
Source: J Nanobiotechnology. 2026 Jan 24;24:169. doi: 10.1186/s12951-026-04049-2 (PMC12910947; doi:10.1186/s12951-026-04049-2)
Supplement: Supplementary file 1 — Supplementary Material 1. [file 12951_2026_4049_MOESM1_ESM.docx]

Supporting Information

Renal-tubular-mitochondrial sequentially targeted nanoagent breaks the vicious cycle of oxidative stress and mtDNA-driven inflammation in acute kidney injury therapy

*Chenli Zhang, Ling Tan, Pengfei Yang, Lili Huang, Zeli Xiang, Linshan Zhao,Ling Zhang*, Jun Deng*, Xiaohui Liao**

**Table S1**. The influence of drug/carrier ratio on DLC and DLE of TMB

| Ratio | 16:1 | 8:1 | 4:1 | 2:1 |
| --- | --- | --- | --- | --- |
| DLC (%) | 4.84±0.04 | 3.94±0.02 | 1.95±0.03 | 2.91±0.06 |
| DLE (%) | 75.37±0.18 | 71.71±0.17 | 77.63±0.2 | 29.06±0.60 |

DLC: drug-loading capacity.

DLE: drug-loading efficiency.


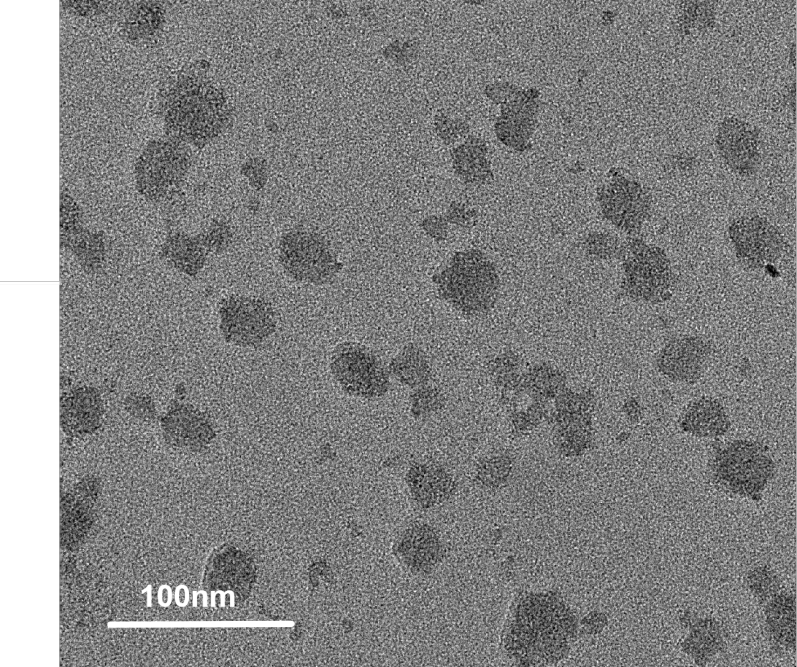


**Figure S1**. Representative TEM image of STMB degradation in 1 mM H₂O₂ for 24 h.


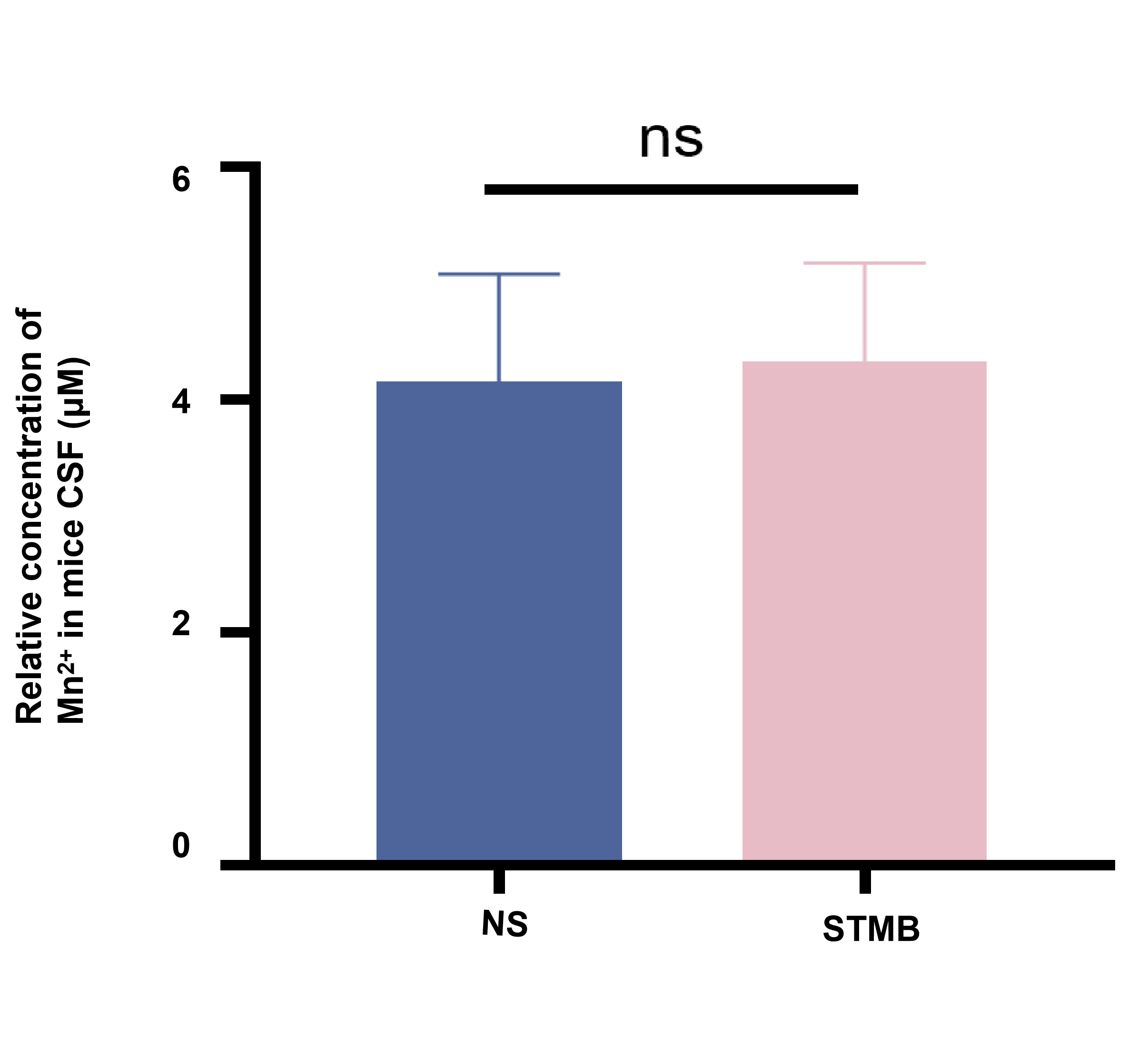


****Figure S2**.The relative concentration of manganese ions in the cerebrospinal fluid（CSF） of mice treated with normal saline or STMB for 12h.** n = 5; *p < 0.05; **p < 0.01; and ***p < 0.001.

**
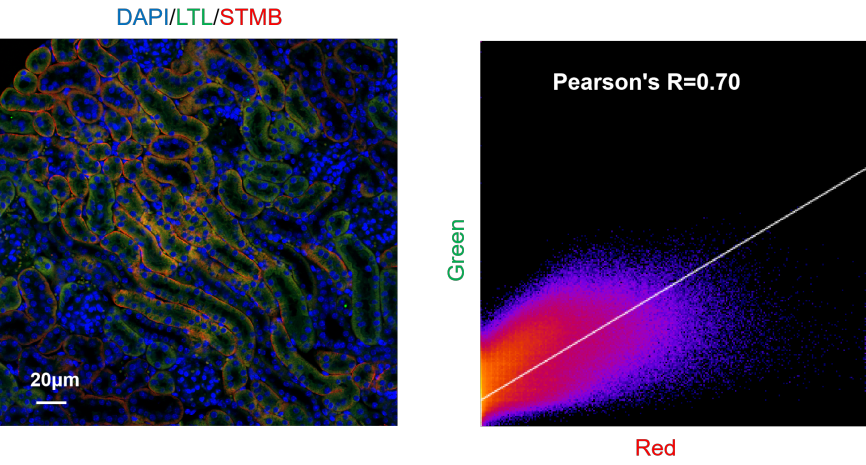
**

****Figure S3**.Pearson correlation analysis between STMB (Cy5-labeled, red) and the proximal renal tubules (LTL-labeled, green).**

**
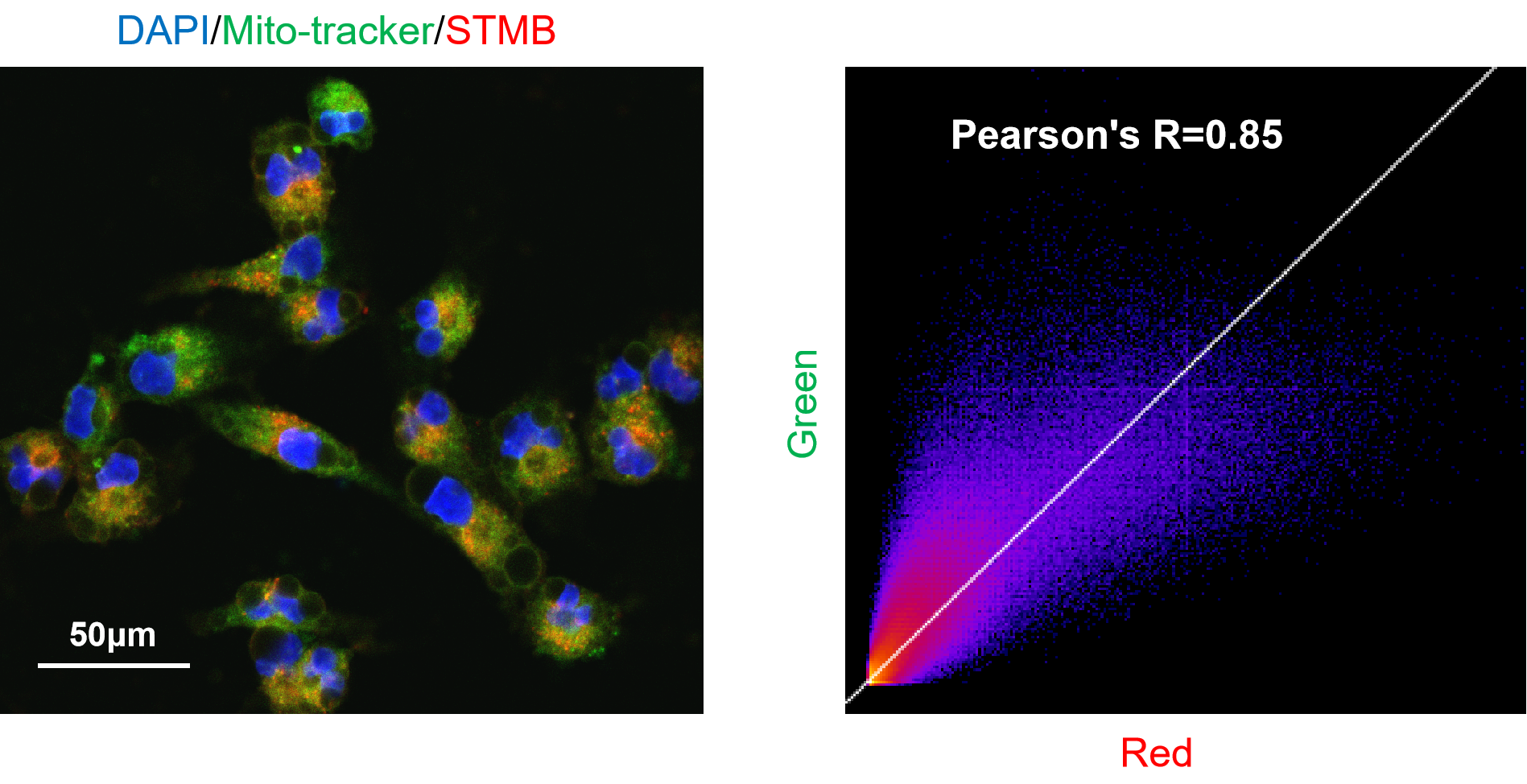
**

****Figure S4**.Pearson correlation analysis between STMB(Cy5-labeled, red) and mitochondria (green).**


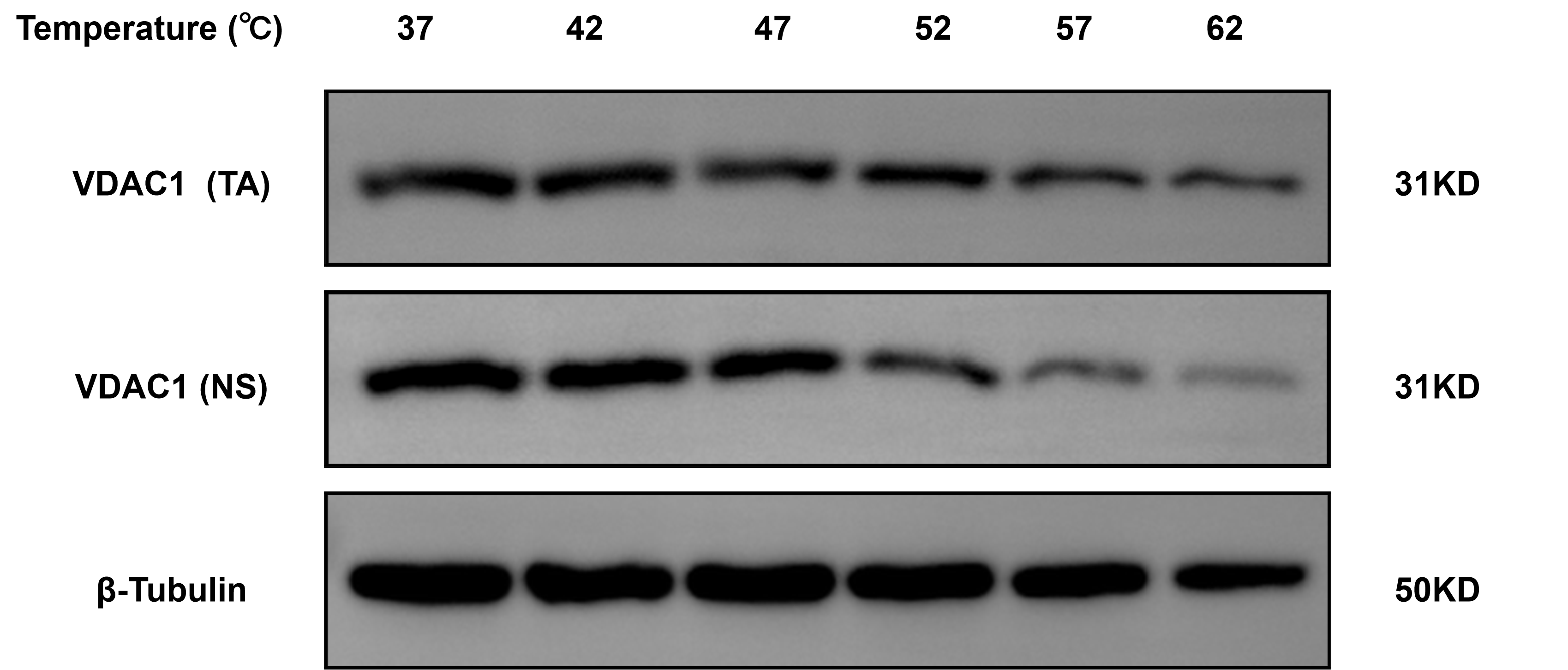


**Figure S5**. Representative Cellular thermal shift (CETSA) images of VDAC1 in HK-2 cells. (Cells were treated with normal saline or TA for 24h )


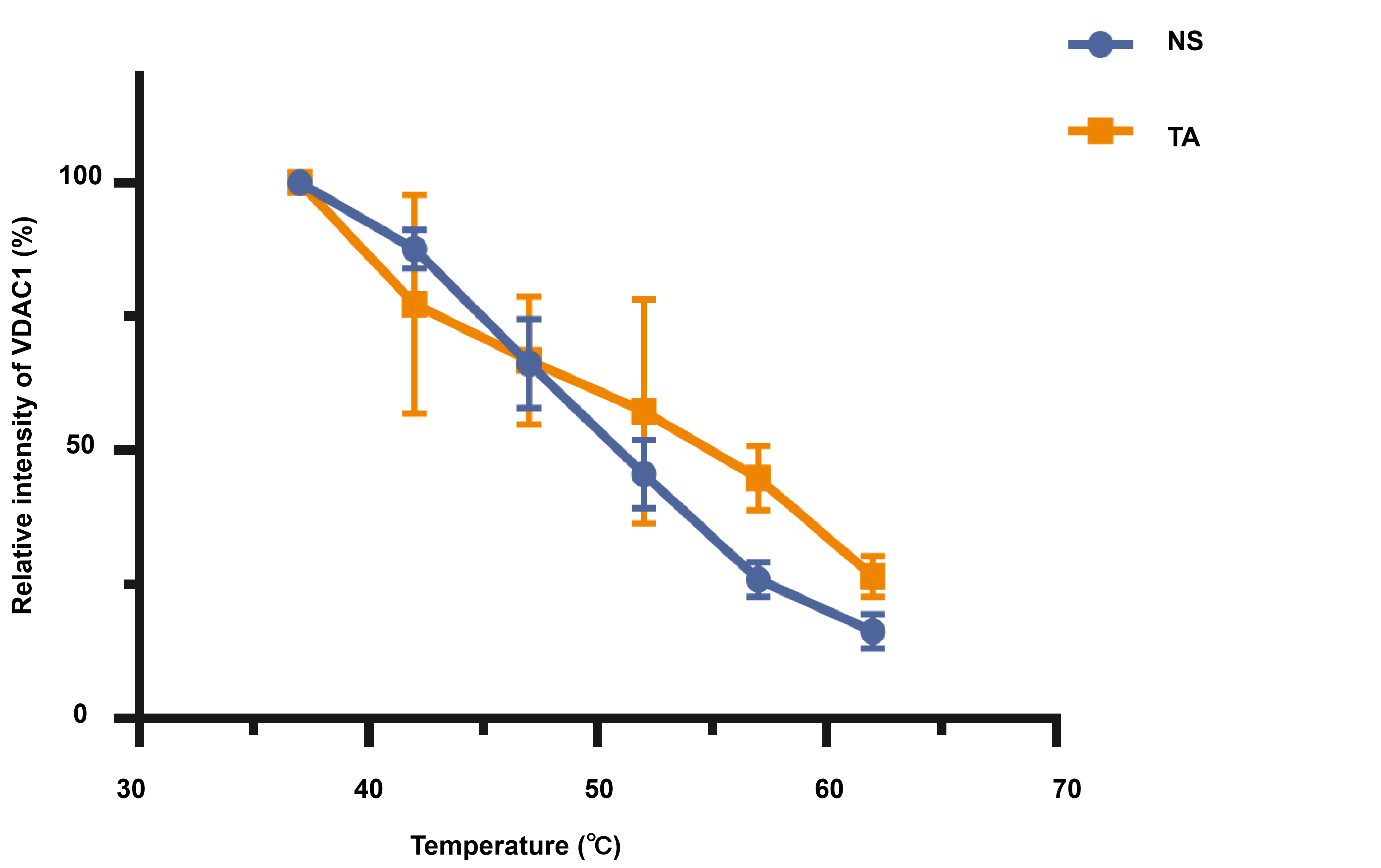


**Figure S6**. Summary of relative intentisity of VDAC1.Data are presented as mean ± SD, n = 3; *p < 0.05; **p < 0.01; and ***p < 0.001.


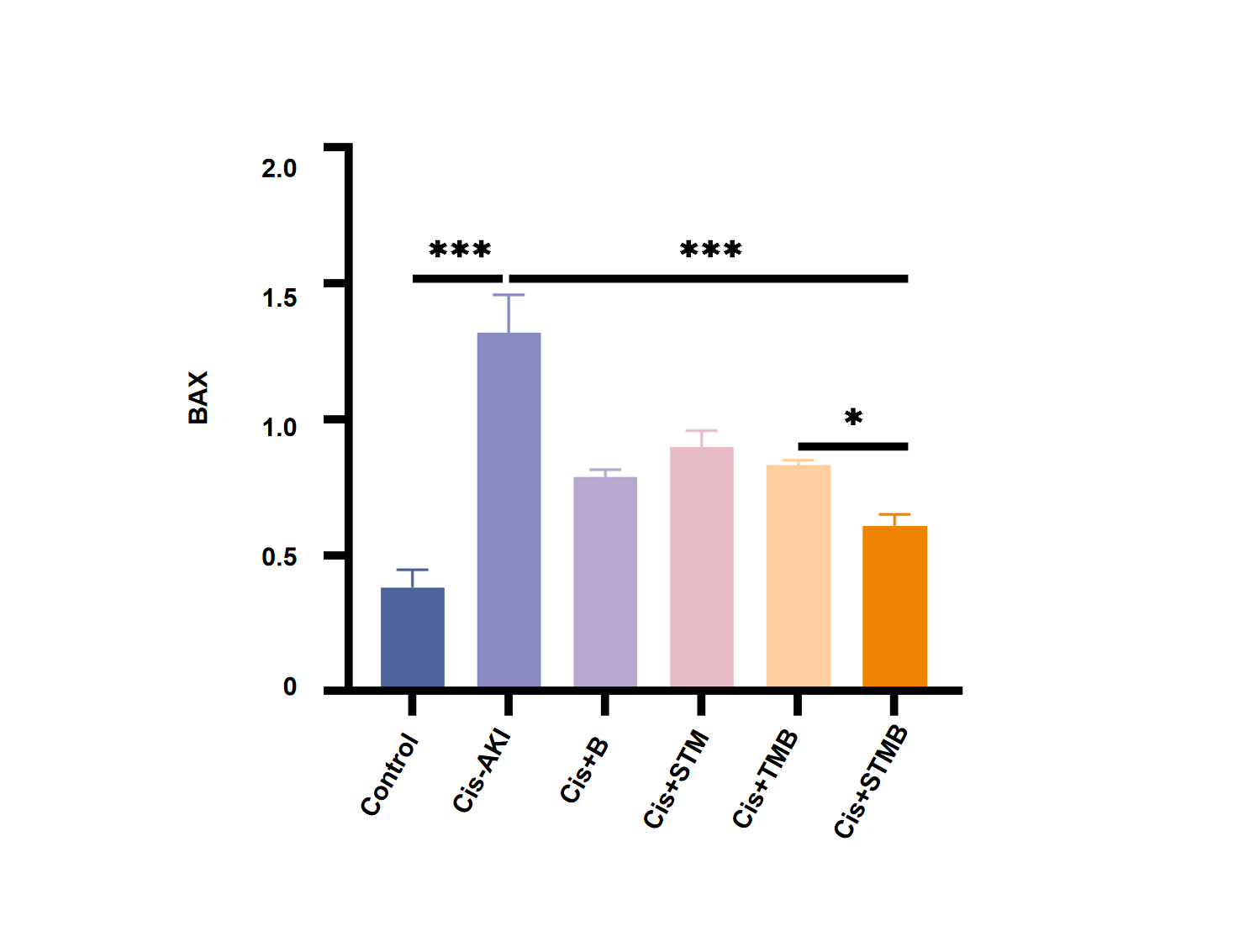


**Figure S7**. Western blot analysis of BAX. β-actin was used to normalize protein content. Data are presented as mean ± SD, n = 3; *p < 0.05; **p < 0.01; and ***p < 0.001.


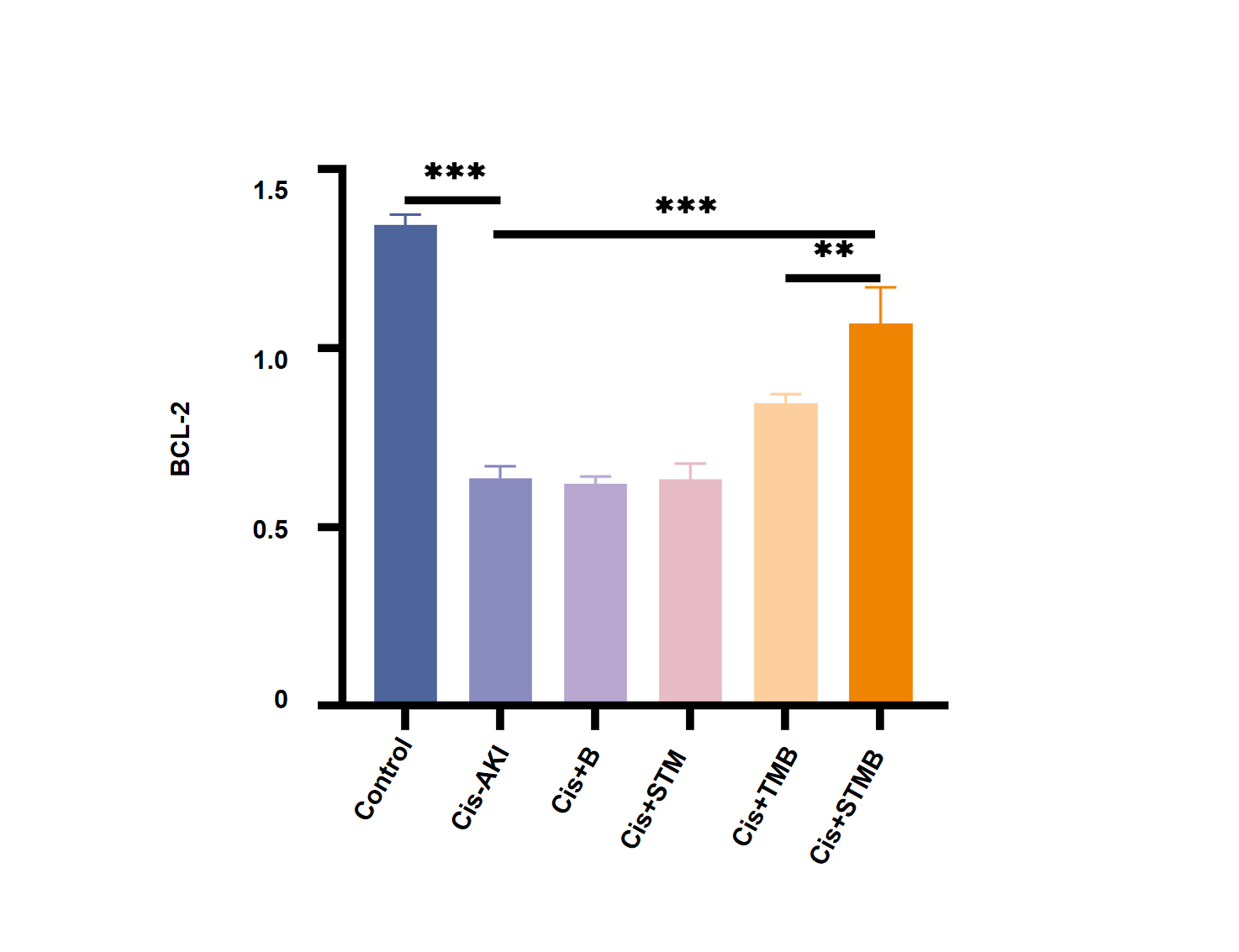


**Figure S8**. Western blot analysis of BCL-2. β-actin was used to normalize protein content. Data are presented as mean ± SD, n = 3; *p < 0.05; **p < 0.01; and ***p < 0.001.


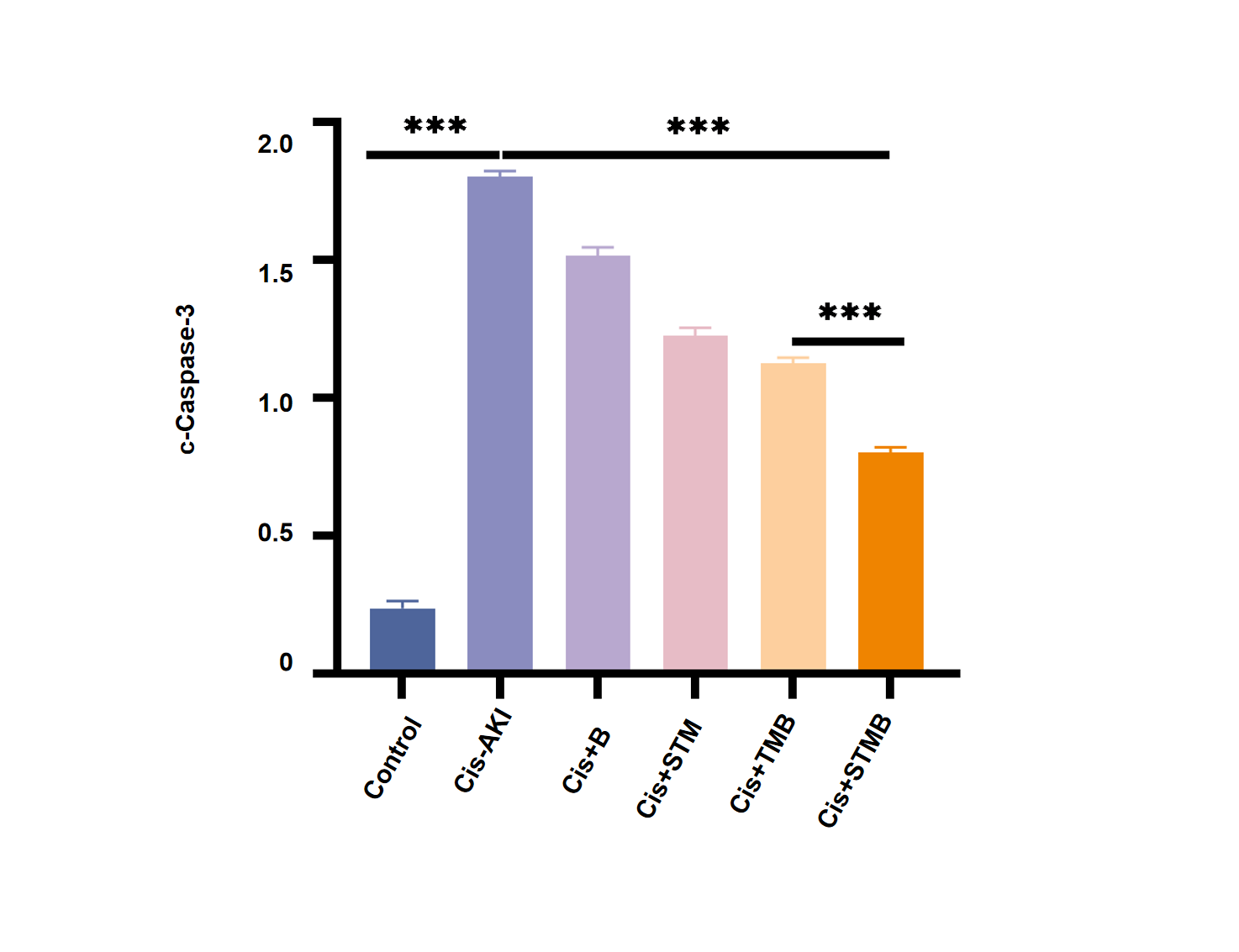


**Figure S9**. Western blot analysis of cleaved-caspase 3. β-actin was used to normalize protein content. Data are presented as mean ± SD, n = 3; *p < 0.05; **p < 0.01; and ***p < 0.001.


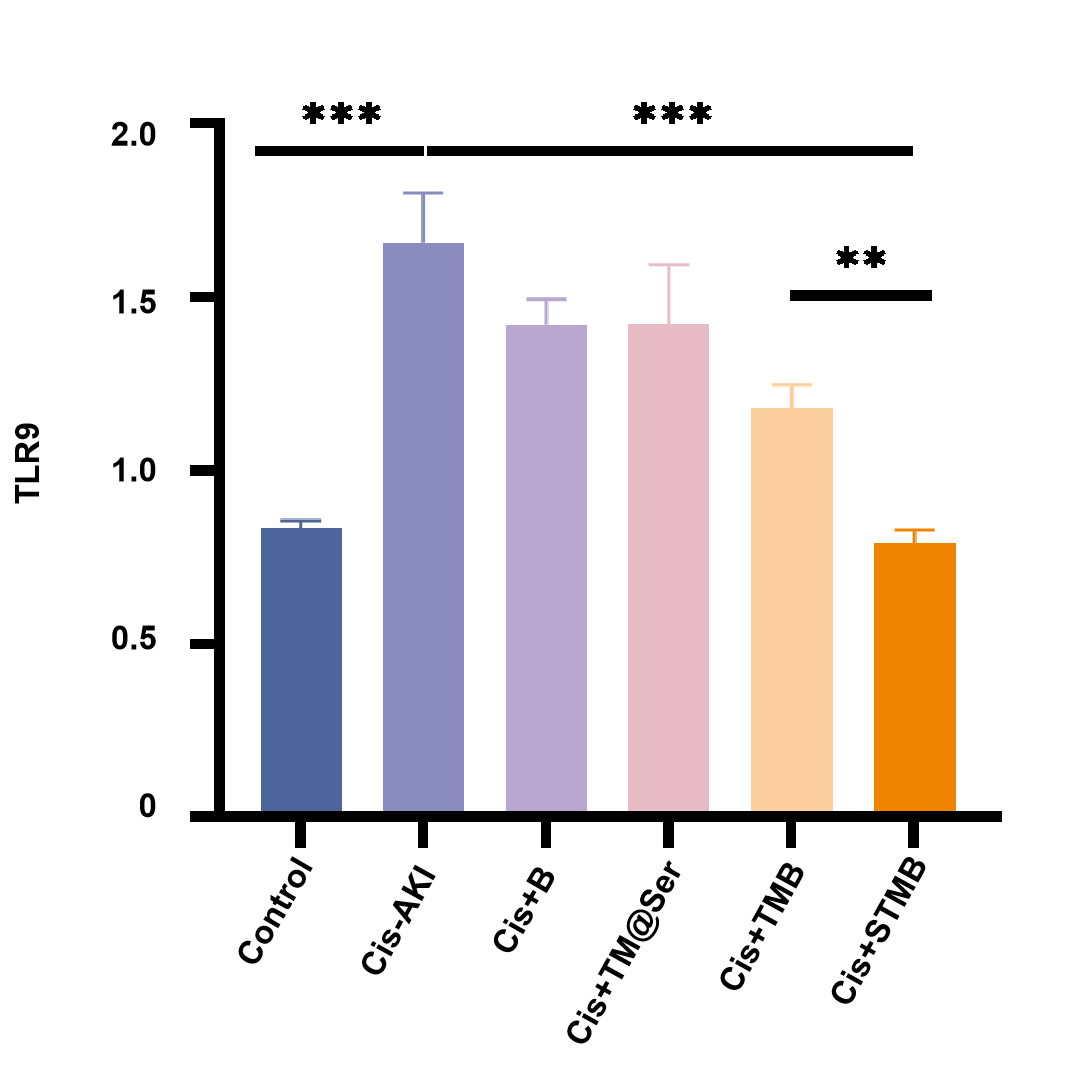


**Figure S10**. Western blot analysis of TLR9. β-actin was used to normalize protein content. Data are presented as mean ± SD, n = 3; *p < 0.05; **p < 0.01; and ***p < 0.001.


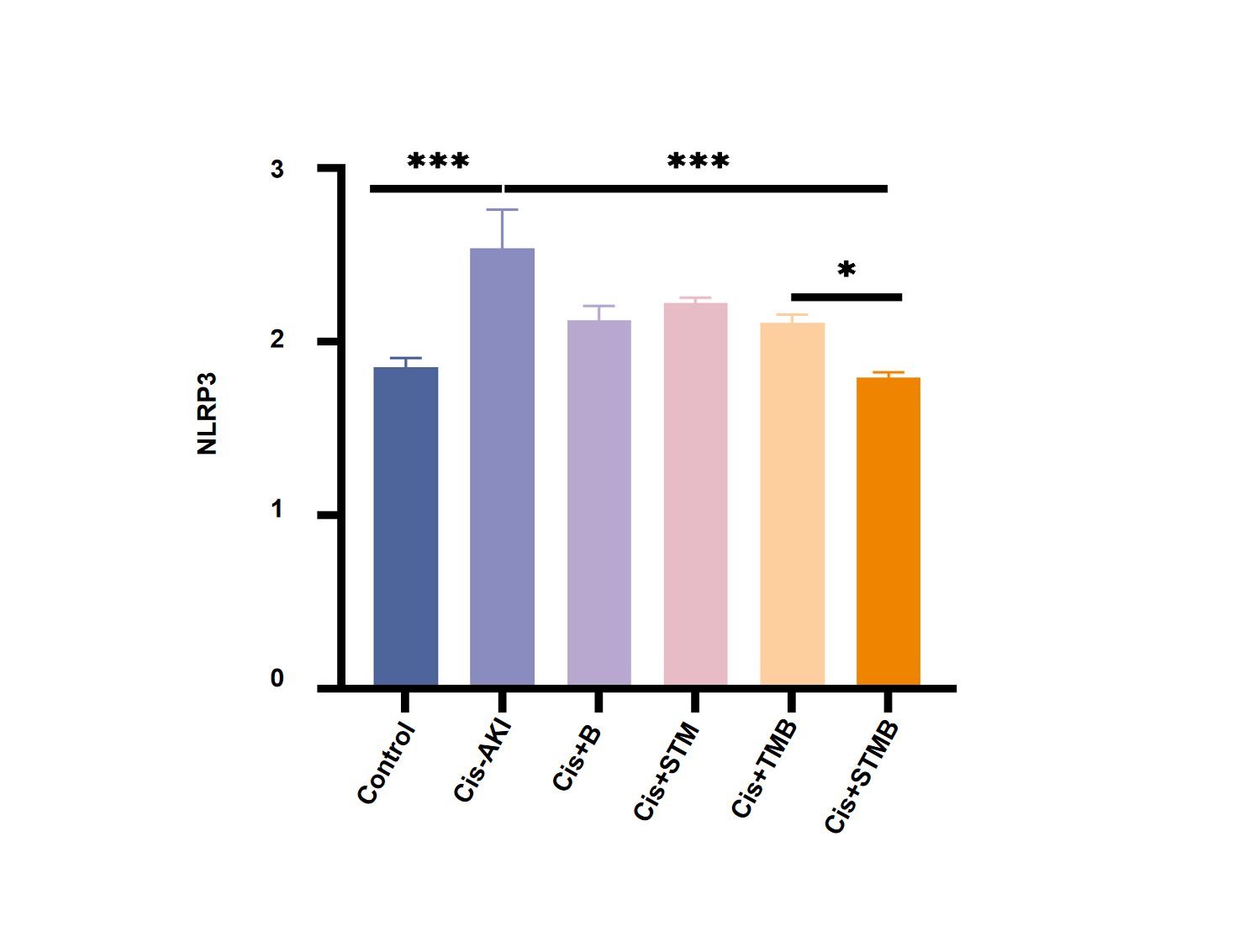


**Figure S11**. Western blot analysis of NLRP3. β-actin was used to normalize protein content. Data are presented as mean ± SD, n = 3; *p < 0.05; **p < 0.01; and ***p < 0.001.


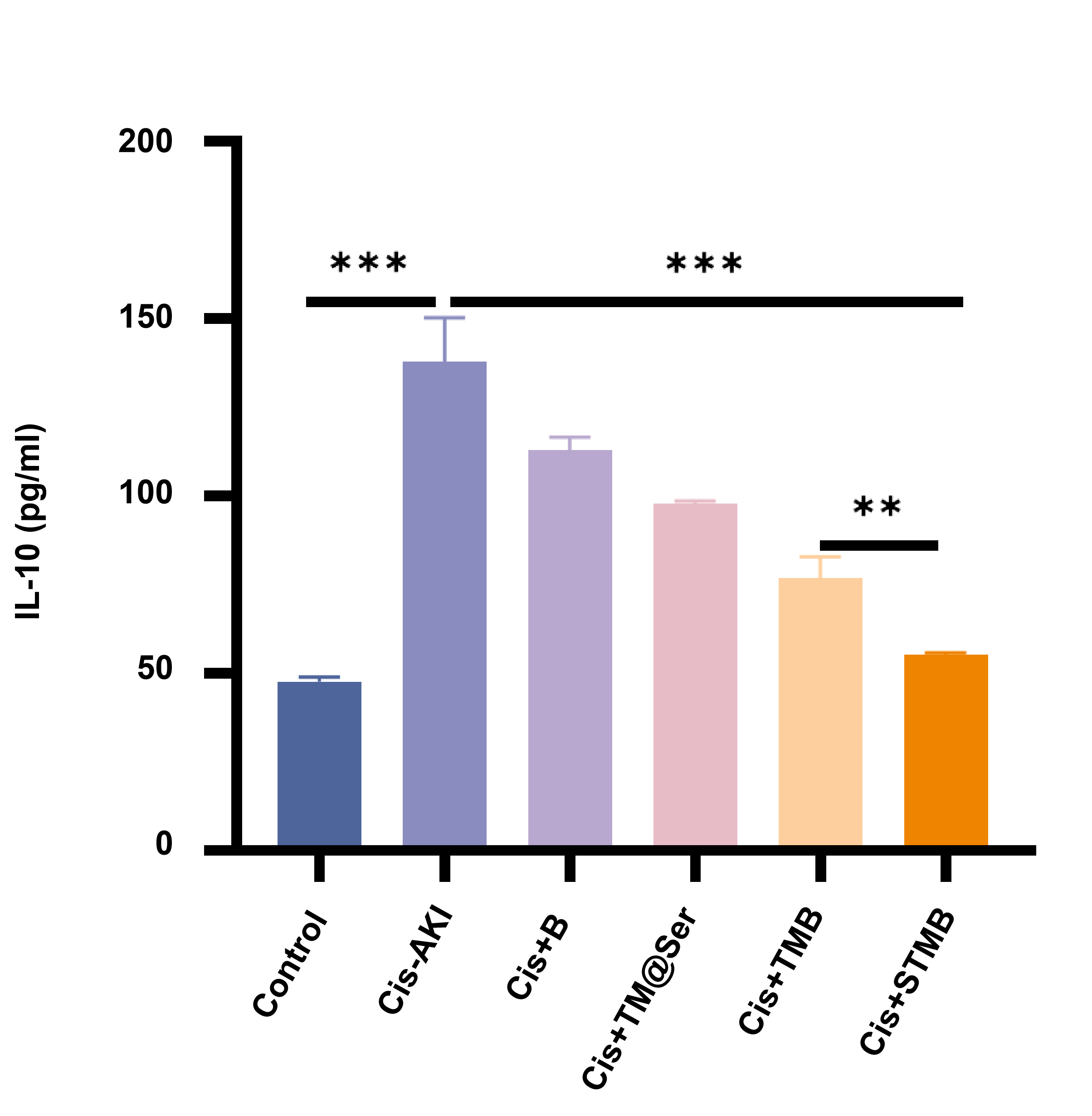


**Figure S12**. Detection of IL-10 levels in mouse serum using ELISA. Data are presented as mean ± SD, n =5; *p < 0.05; **p < 0.01; and ***p < 0.001.


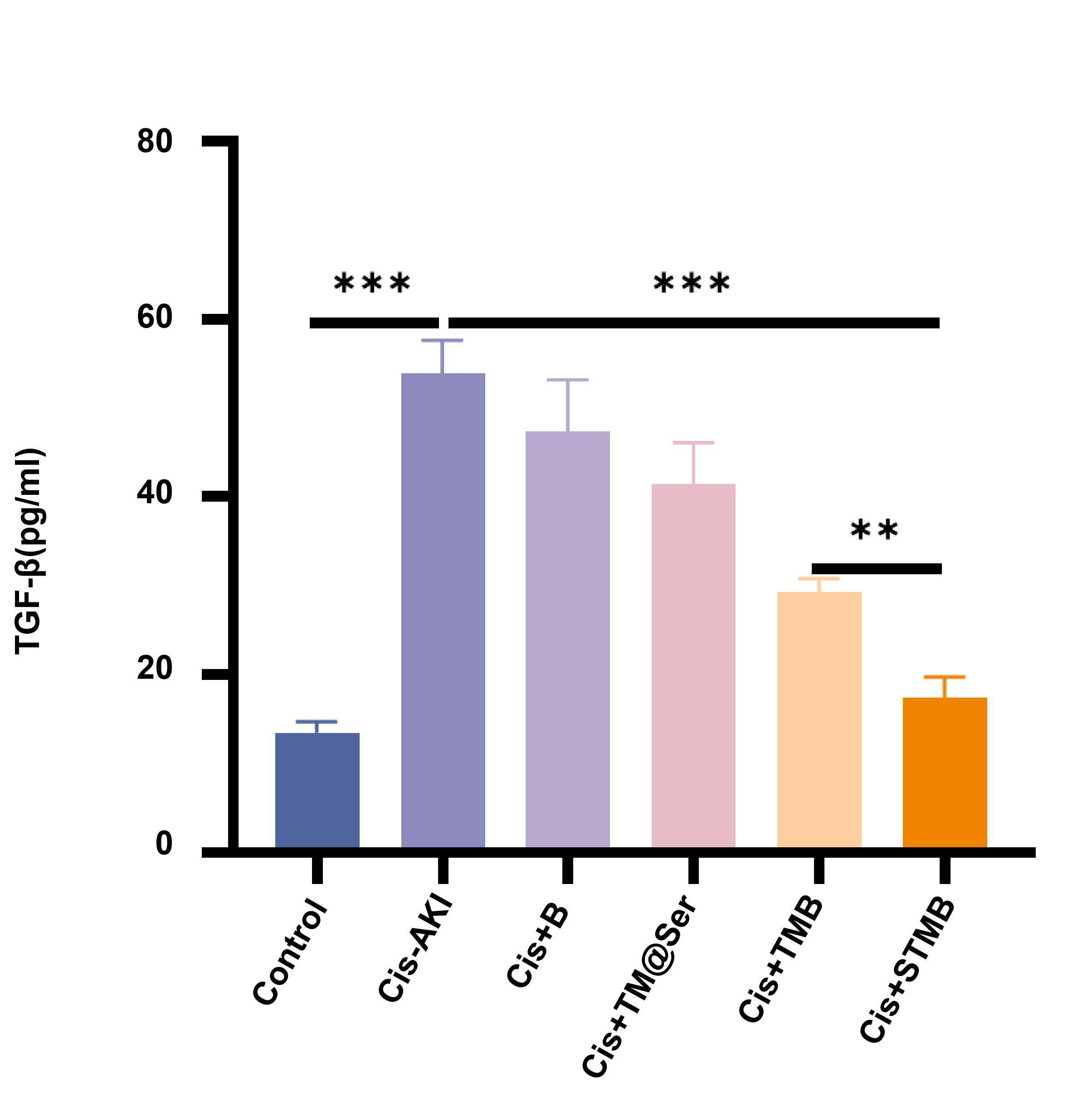


**Figure S13**. Detection of TGF-β levels in mouse serum using ELISA. Data are presented as mean ± SD, n =5; *p < 0.05; **p < 0.01; and ***p < 0.001.


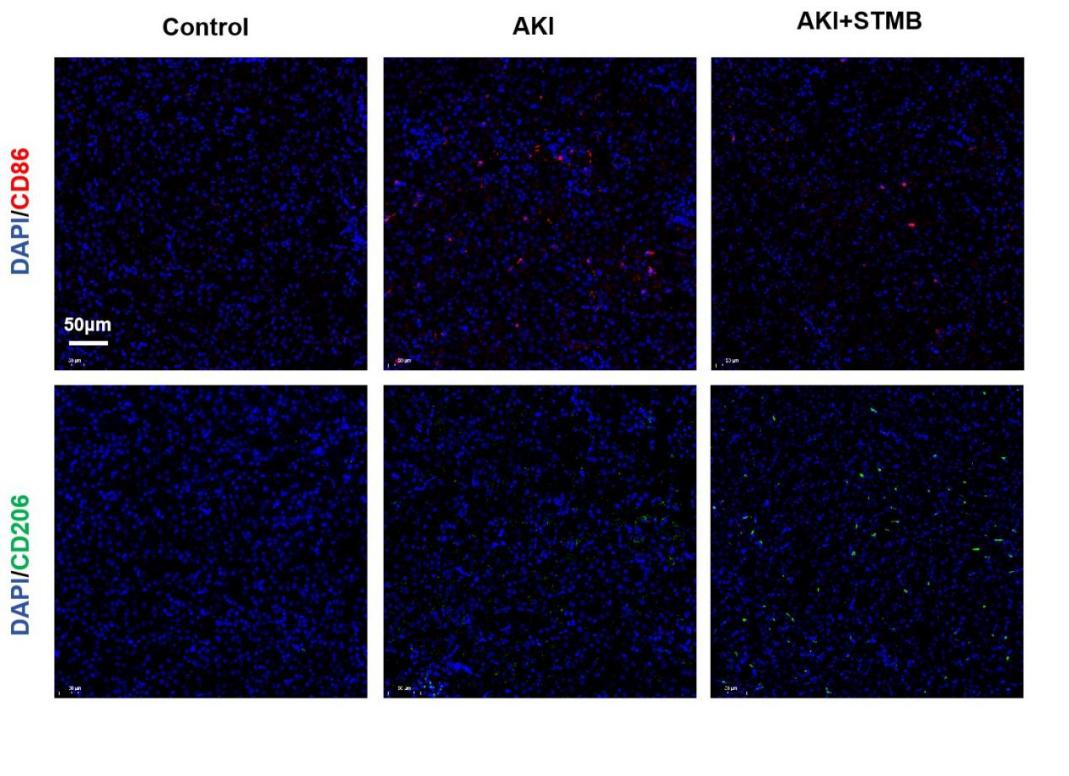


**Figure S14**. Representative immunofluorescence images of kidneys from each group showing CD86 (red), CD206 (green), and DAPI (blue).
